# Robust adaptive PD-like control of lower limb rehabilitation robot based on human movement data

**DOI:** 10.7717/peerj-cs.394

**Published:** 2021-02-24

**Authors:** Ningning Hu, Aihui Wang, Yuanhang Wu

**Affiliations:** 1 School of Mechatronic Engineering and Automation, Shanghai University, Shanghai, China; 2 School of Electric Information Engineer, Zhongyuan University of Technology, Zhengzhou, China; 3 School of Materials Engineering, Shanghai University of Engineering Science, Shanghai, China

**Keywords:** Human movement data, Rehabilitation robot, Trajectory tracking, S-curve function, Similarity function

## Abstract

The combination of biomedical engineering and robotics engineering brings hope of rehabilitation to patients with lower limb movement disorders caused by diseases of the central nervous system. For the comfort during passive training, anti-interference and the convergence speed of tracking the desired trajectory, this paper analyzes human body movement mechanism and proposes a robust adaptive PD-like control of the lower limb exoskeleton robot based on healthy human gait data. In the case of bounded error perturbation, MATLAB simulation verifies that the proposed method can ensure the global stability by introducing an S-curve function to make the design robust adaptive PD-like control. This control strategy allows the lower limb rehabilitation robot to track the human gait trajectory obtained through the motion capture system more quickly, and avoids excessive initial output torque. Finally, the angle similarity function is used to objectively evaluate the human body for wearing the robot comfortably.

## Introduction

Patients with lower limb movement disorders are usually caused by neurological diseases, such as stroke, hemiplegia, and others (Yang et al., 2017). The traditional effective rehabilitation methods are medical rehabilitation doctors and drug treatment. One problem of this approach is that due to the population aging, the increasing rate in the number of patients exceeds the capacity medical personnel for the assistive function training for all patients with motor dysfunction (Kim et al., 2020; Kim & Lee, 2019). The emergence of exoskeleton robots, including those virtual haptic system (Wang et al., 2020; Jiang et al., 2017), based on robotic systems and biomedical engineering principles has brought good news to patients, providing effective rehabilitation for patients without special care (Badesa et al., 2016; Chen et al., 2019). It is the mission of the lower limb rehabilitation robot (LLRR) to enable the patient's lower limbs to move or walk like a healthy person through the exoskeleton robot rehabilitation training. Therefore, the gait motion data of the human body can be used in the design and real-time training of the human-like controller of the LLRR after optimization processing. In the field of human sports rehabilitation technology, the measurement of the body movement process is one of the current challenges. Detailed movement information can be used for the verification of biomechanical models, and it is significant for human body dynamics analysis (Abd et al., 2020). The human body is a complex structure with multiple degrees of freedom of rotation joints. Generally, the motion and motion trajectory of the human body are recorded through motion capture technology. After analyzing the captured data, a lot of information about the human body at a certain moment can be accurately obtained, such as spatial position, velocity, acceleration, angle, angular velocity, angular acceleration, and so on (Liu et al., 2017).

The LLRR is a highly complex mechatronics system and its dynamic model is a multi-input, multi-output, strongly coupled nonlinear differential equation with many uncertainties (Yin et al., 2019; Becerra et al., 2018; Zhang et al., 2016). When patients undergo rehabilitation training, LLRR is required to have good positioning accuracy and fast trajectory tracking capabilities, so many dynamic control schemes based on dynamic models have been proposed (Yang et al., 2020; Zhang, Hu & Gow, 2020; Ariizumi et al., 2019). The dynamic control scheme based on the dynamic model includes modern control methods such as adaptive control (Ling, Wang & Liu, 2020; Lee et al., 2019; Cheng et al., 2011), and robust control (Yang et al., 2020; Saeed & Qin, 2019; Xue et al., 2019, Cheng et al., 2012). There are also intelligent control methods such as fuzzy control (Ling, Wang & Liu, 2020; Kong et al., 2019, 2018) and neural network control (Li et al., 2016). These control methods can solve some of the system's own nonlinearity, coupling, external interference and other uncertain problems, and improve the robustness and control accuracy of the system. However, the dynamic control scheme based on the dynamic model of the LLRR requires a large amount of real-time online calculation of dynamics, which brings difficulties to the dynamic real-time control of the LLRR. Because the LLRR system is a highly complex multi-variable, strongly coupled nonlinear system. Therefore, when designing an LLRR controller, variables are usually introduced to transform a nonlinear system into a linearized system, according to the dynamic characteristics of the LLRR system. This method simplifies the LLRR system and satisfies robust stability. For example, Boudjedir, Boukhetala & Bouri (2018) proposed a nonlinear proportional derivative control (NPD) to solve the trajectory tracking problem of robot. Although this method is simple and easy to implement, it has poor resistance to external interference and requires Larger driving torque, which is difficult to apply in robot systems. Cao, Zhang & Zhu (2017) raised a nonlinear compensation control algorithm in case of combining PID control with an active disturbance rejection control. The control strategy improves control accuracy and robustness of multi-degree-freedom industrial robot at high speeds. The geometric parameters of the LLRR system are known or can be accurately measured, but its inertial parameters are often inaccurate or completely unknown. A variable impedance strategy based on an uncertainty and disturbance estimator is proposed (Dong & Ren, 2019), which improves the adaptability and anti-interference of uncertain robot system perform tasks in an uncertain environment. The uncertainty and disturbance estimator is mainly used to deal with unknown environmental changes. To solve the problem of non-linear and uncertain system equipment with external interference, Celentano & Basin (2020) provided an approach to design robust smooth controller. A sufficiently smooth reference signal can be tracked well when an error norm smaller than a prescribed value. Therefore, Xue et al. (2019) proposed an adaptive PI controller based on the dynamic model with perturbations and uncertainties is presented for trajectory tracking control, and its stability analysis is given via the Lyapunov Theory. However, it is particularly important for the comfort and subjectivity of the patient's rehabilitation training, the convergence speed and accuracy of trajectory tracking when designing the controller for the LLRR. At present, most researches on trajectory tracking control are discussed sine waves as input or gait trajectory planning, which has a certain significance for the research of controllers, but it cannot satisfy patients with lower limb movement disorders to carry out effective rehabilitation training.

**Figure 1:**
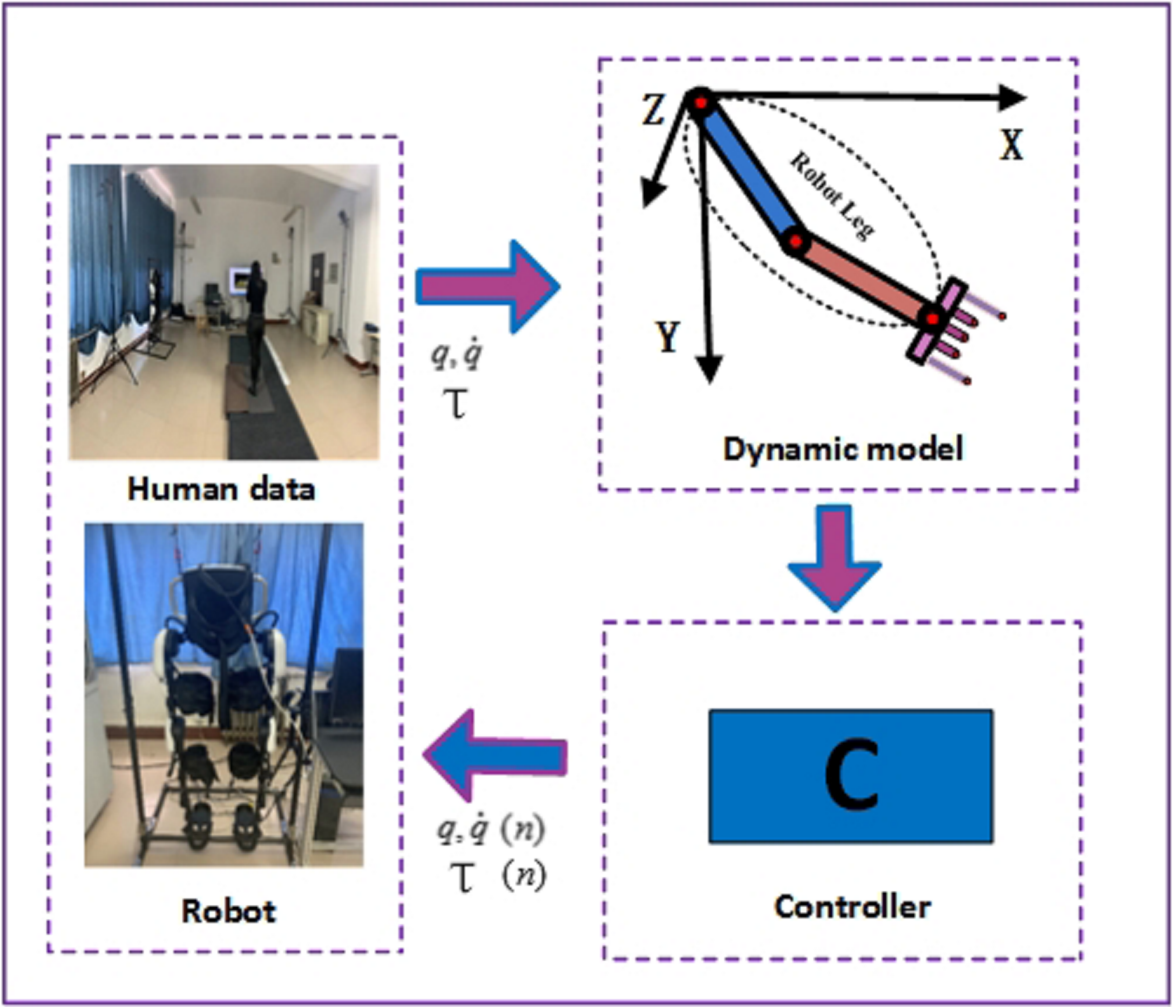
The schematic diagram of control principle.

Therefore, this paper adopts an nonlinear complex exoskeleton robot as the research object, and designs a robust adaptive PD-like control (RAPLC) based on real data. The schematic diagram of the principle is shown in Fig. 1. First, NOKOV three-dimensional (3D) infrared motion capture equipment and three-dimensional force measurement platform equipment are used to collect human gait movement data and plantar force data. The collected data is analyzed for human kinematics and dynamic characteristics and the processed gait data is used as the reference expected motion trajectory of the LLRR control system. Second, the S-curve function is introduced on the basis of the traditional robust adaptive PD controller, which improves the convergence speed of the joint angular displacement and reduces the joint initial torque. Finally, the quadratic function of the error is used as the energy function of the system, and the stability of the control system of the LLRR based on the Lyapunov stability theory is guaranteed under the condition of the coupling and nonlinearity of each joint. The trajectory tracking simulations results show that the LLRR system joint angular displacement has good tracking performance. Comparing the LLRR system joint torque obtained from simulation with experimenter’s lower limbs can safely and comfortably complete the rehabilitation training.

### Analysis of the mechanism of human lower limb movement

#### Human lower limb movement data collection

**Figure 2:**
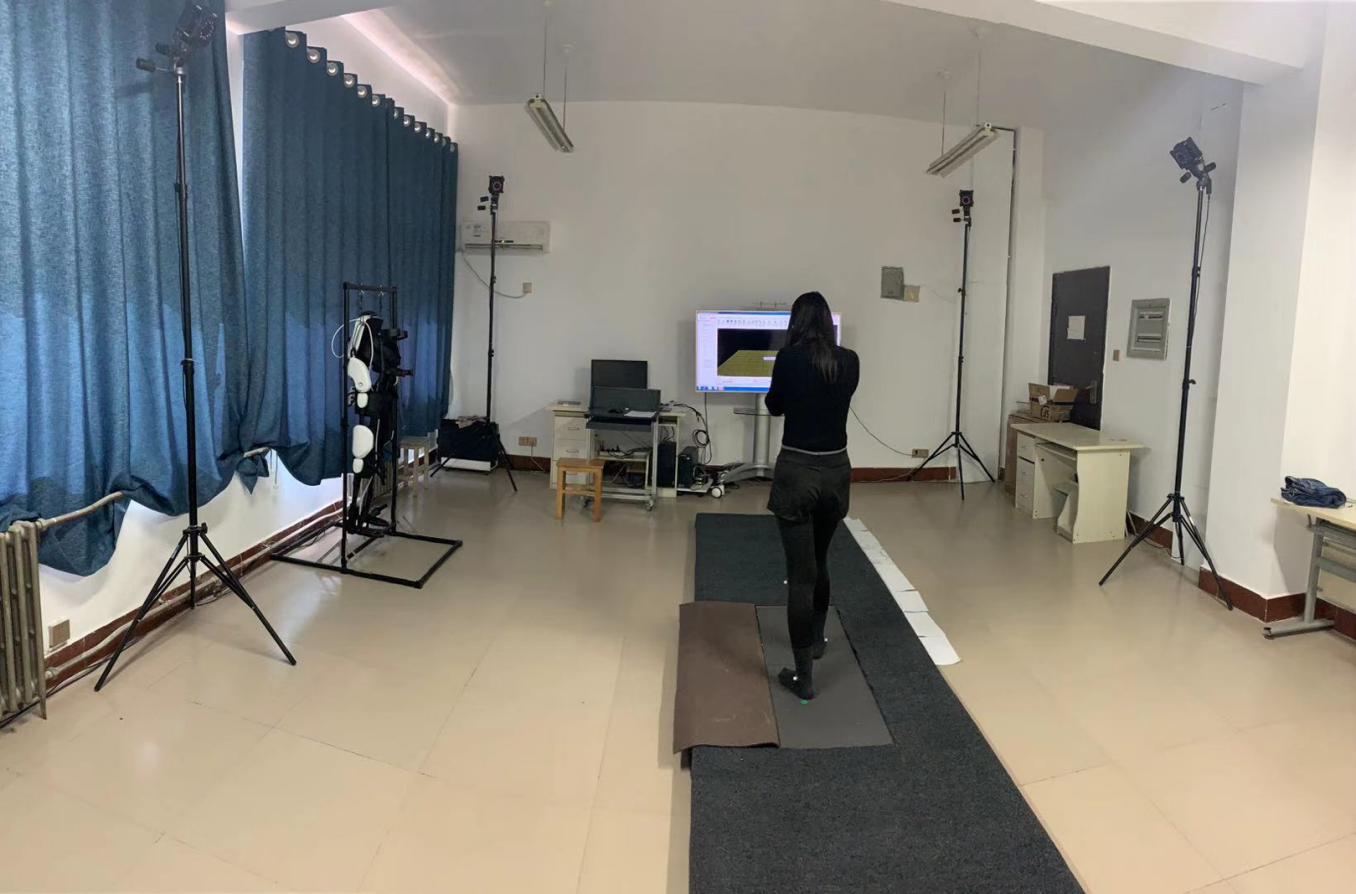
Motion capture scene.

The existing motion capture technology is mainly divided into four categories: mechanical motion capture system (Ivanov & AZhilenkova, 2019), acoustic motion capture system, electromagnetic motion capture system, and optical motion capture system (Ren et al., 2018; Pfister et al., 2014). Through the comparison of motion capture systems, this paper adopts the better NOKOV 3D infrared passive optical motion capture system. This system work principle is to capture the movement trajectory of the reflective Marker point attached to the human body in space by using passive infrared light principle. The 3D force measurement platform system mainly realizes the mechanical force measurement and analysis functions of the human body during the movement process. During the measurement, the force in the *x*, *y* and *z* axes are integrated into multiple channel data through 3D force sensors, and then collected by NI Card. Before collecting experimental data, site layout is essential. The cameras of the motion capture system are usually arranged according to the experimental requirements (Kurihara et al., 2002; Zeng et al., 2016). Because the experiment done in this paper is to collect human lower limb movement data, cameras are placed in a spac with 5 × 4 × 3 m. The cameras are placed in three equal intervals in each column on the left and right, and the 3D force measurement platforms are placed in the center, as shown in Fig. 2.

The NK_Cortex software can observe the movement state of the Marker points attached to the human body in real time. When the human body moves in the space of the motion capture system, as long as the Marker points at the joints can be captured by two or more cameras at the same time, the location information of the Marker points is recorded. Store the collected human movement data in real time and save it in required files format through the software. The quality of data collection plays an important role in the research. Therefore, after collecting the data, it is necessary to check whether the position, velocity, acceleration and other information of each Marker point are missing, and observe whether the curve is disconnected. If there is an obvious disconnection in the middle of the curve means that the data of the frame position is lost. If the lost data fluctuates greatly or the number of consecutive lost frames is large, data collection needs to be repeated, otherwise the data can be repaired.

#### Gait trajectory data processing

The collected human gait accuracy will affect the stability, coordination and driving torque of LLRR system. The ideal gait is the continuous and smooth motion trajectory of each joint, such as position, angle, angular velocity and angular acceleration. The 3D motion gait capture system mainly captures the spatial position coordinate information of the hip joint, knee joint and ankle joint. During the measurement process, NK_Cortex software can repair the curve disconnection problem, such as the missing coordinate data of some Marker points due to occlusion or other external factors. However, there are external factors such as marker jitter and environmental capture during human movement, spliced data needs to be filtered at this time. Filtering methods mainly include Least Squares filtering, Wavelet filtering, Kalman filtering, and others. In this paper, least square filtering and wavelet filtering are used to process the data of human left leg hip joint, knee joint and angle joint.

**Figure 3:**
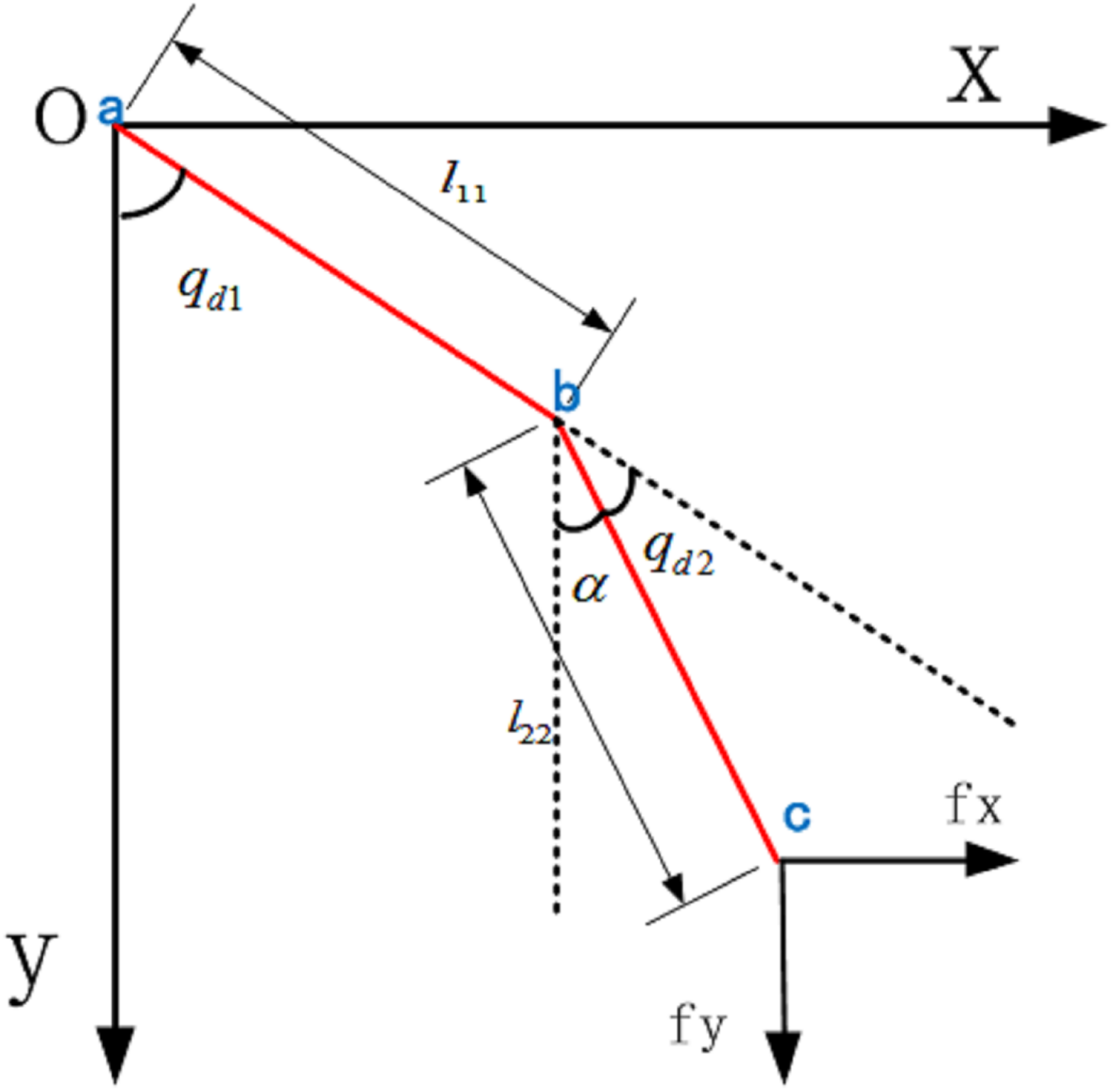
LLRR-like model.

The kinematics of the robot is used to study the geometric relationship between the motion angle of each joint of the LLRR and its position in space. Similarly, the relationship between the spatial position coordinates of each joint of the human body's lower limb and the angle of each joint is found through the collected data, which plays a vital role in the control of the gait trajectory data and the joint angle of the LLRR in the later stage. Kinematics is divided into positive kinematics analysis method and inverse kinematics analysis method. Based on the needs of experimental research, the LLRR-like mode as shown in Fig. 3. this paper adopts the inverse kinematics analysis method, that is, using the position data of the end of the human lower limb hip joint $a\left( {{a_x},{a_y}} \right)$, knee joint $b\left( {{b_x},{b_y}} \right)$ and ankle joint $c\left( {{c_x},{c_y}} \right)$, the hip joint angle ${q_{\rm d1}}$ and knee joint angle ${q_{\rm d2}}$ can be obtained through the tangent theorem,

(1) $${q_{\rm d1}} = \arctan \displaystyle{{{b_x} - {a_x}} \over {{b_y} - {a_y}}}$$

(2) $${\rm{\alpha}} = \arctan \displaystyle{{{c_x} - {b_x}} \over {{c_y} - {b_y}}}$$

(3) $${q_{\rm d2}} = {q_{\rm d1}} - {\rm{\alpha}} = {q_{\rm d1}} - \arctan \displaystyle{{{c_x} - {b_x}} \over {{c_y} - {b_y}}}$$

From the results obtained from the above two formulas and analyzing the normal gait of the human body during walking, it can be obtained ${q_{\rm d1}} \in \left[ {\matrix{ 0 & {\textstyle{\pi \over 2}} \cr } } \right]$, ${q_{\rm d2}} \in \left[ {\matrix{ 0 & {\textstyle{\pi \over 2}} \cr } } \right]$. Therefore, the inverse kinematics equations of the hip and knee joints of the human body can be obtained as,

(4) $$\left\{ {\matrix{ {{q_{\rm d1}} = \arctan \displaystyle{{{b_x} - {a_x}} \over {{b_y} - {a_y}}}} \cr {{q_{\rm d2}} = {q_{\rm d1}} - \arctan \displaystyle{{{c_x} - {b_x}} \over {{c_y} - {b_y}}}} \cr } } \right.$$

**Figure 4:**
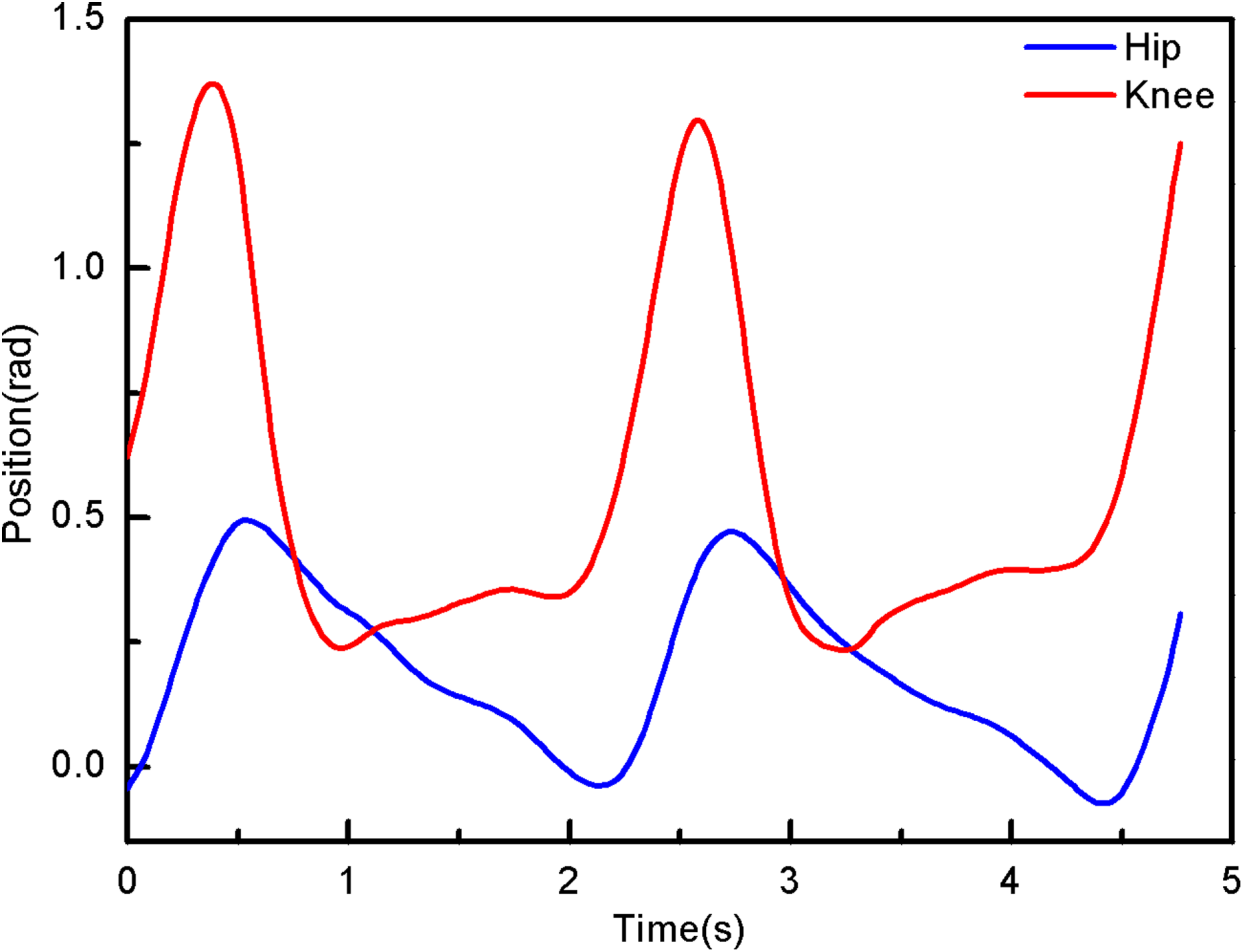
The angle of hip and knee joint.

Its movement trajectory is shown in Fig. 4.

#### Force data processing

Since the rhythm of the human body during walking is not fixed, under-sampling or over-sampling will occur when the movement is too fast or too slow in the process of force data acquisition (Xia et al., 2017; Zhou et al., 2020). Over-sampling will result in excessive information collection, which increases the burden of data analysis, and low utilization. Under-sampling will cause some essential information lost (Zhang & Cao, 2015). Therefore, this paper uses machine learning methods to process the collected 100 sets of force data. In order to deal with data over-sampling, the cubic spline interpolation method is used for processing, and the final processed data is 128 frames per second.

**Figure 5:**
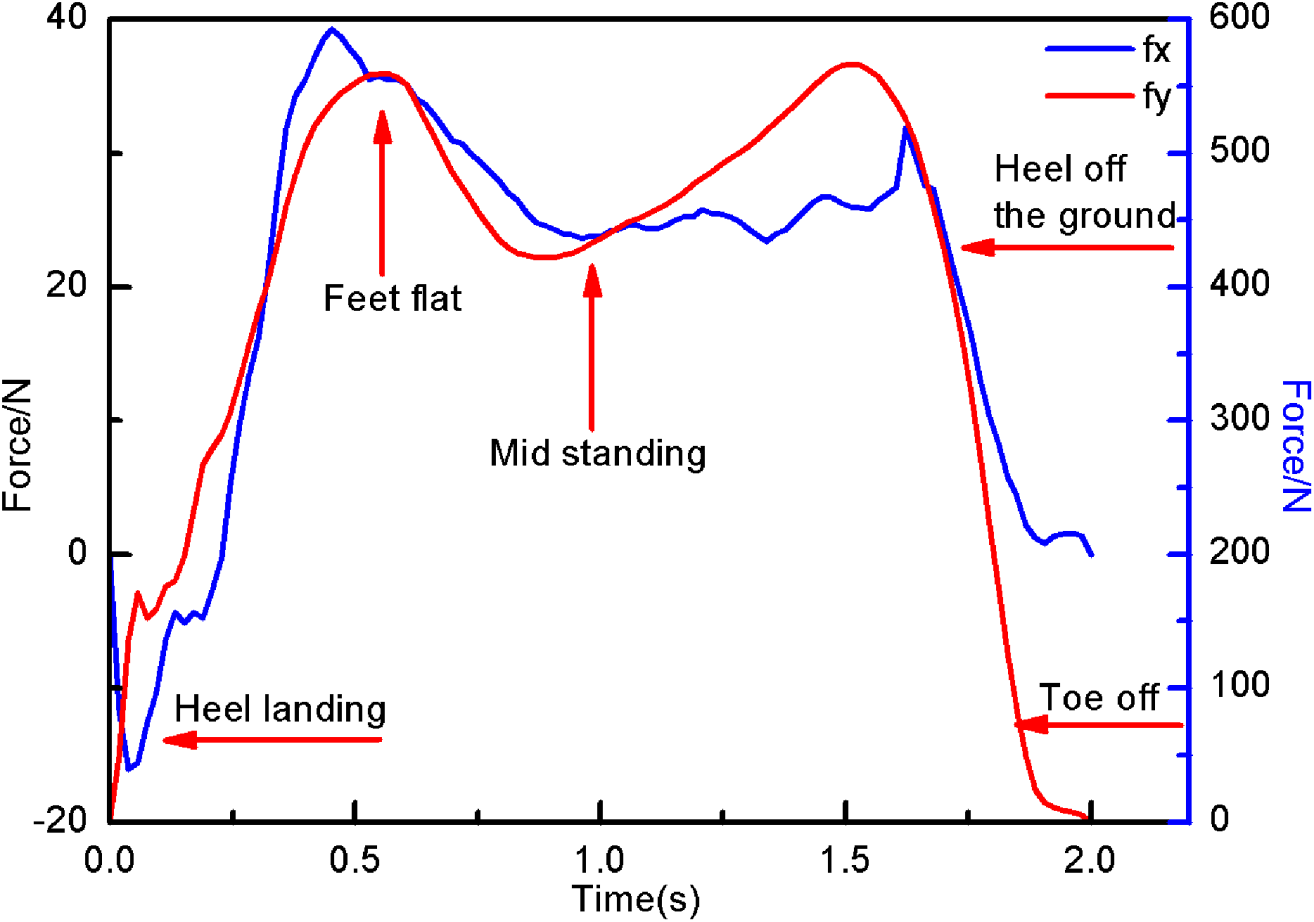
The torque in the x and y directions of c.

The 3D force measurement platform system collects the 3D force data of the end position of the experimenter’s lower limbs. This paper research is based on a two-dimensional plane, so the force in the *x* and *y* directions are selected, as shown in Fig. 4. And the force component of the position *c* can be calculated is shown in Fig. 5.

The force in the *x* direction is,

(5) $${M_x} = {f_x}({c_y} - {a_y})$$

The force in the *y* direction is,

(6) $${M_y} = {f_y}\left( {{c_x} - {a_x}} \right)$$

where, ${f_x}$, ${f_y}$ respectively present the component on the *x*-axis and *y*-axis during real-time change of the collected experimentation three-dimensional plantar force.

The Jacobian matrix $J$ of LLRR system establishes a mapping from joint speed to operating, which can better analyze the speed of system. Similarly, ${J^T}$ represents the force Jacobian matrix, which means a mapping linear relationship between operating force and joint force of LLRR system in the static equilibrium state.

**Figure 6:**
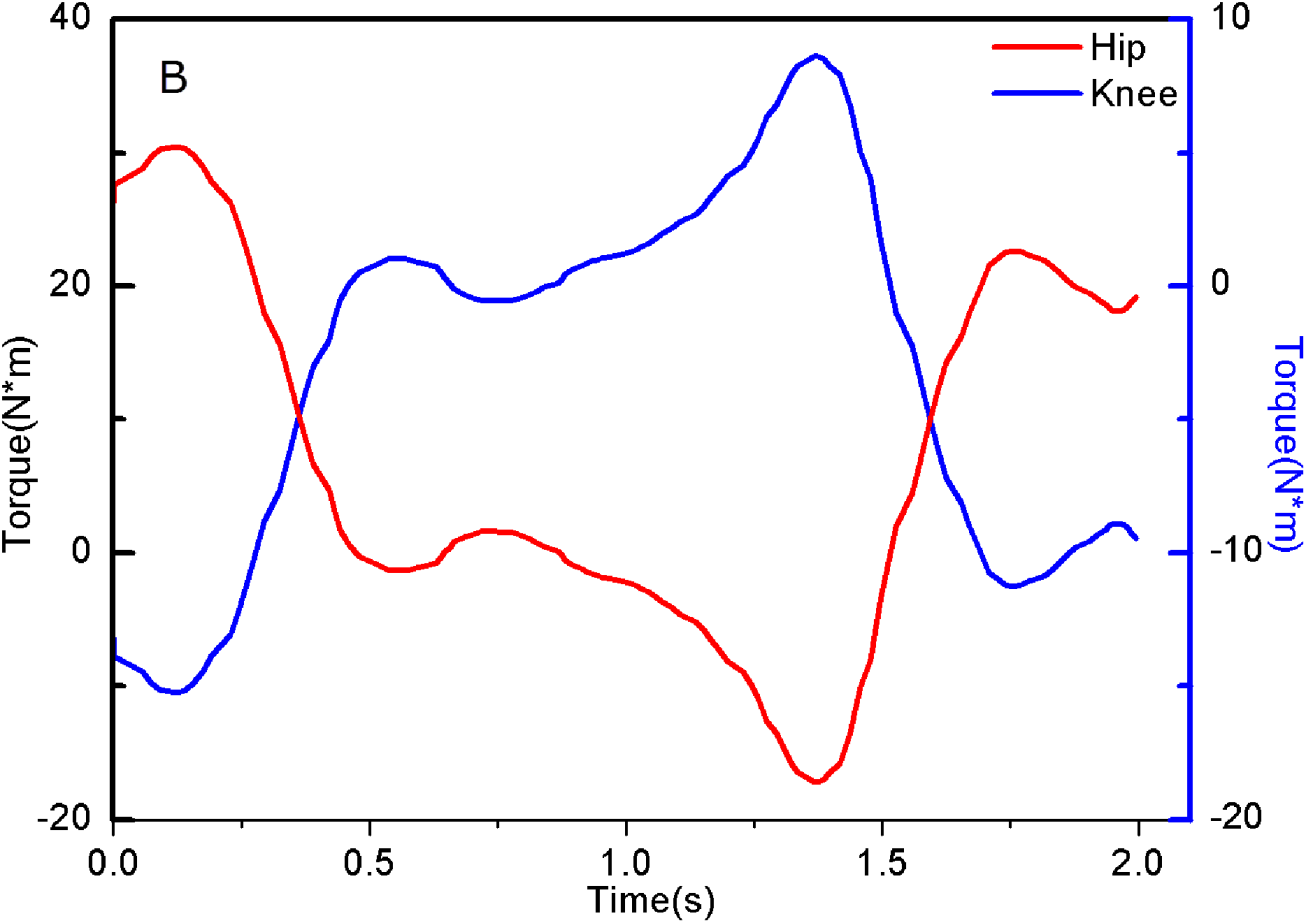
Hip and Knee joint Torque of experimenter.

The torque of the hip and knee joint positions can be obtained by the following equations, and its changes are shown in Fig. 6,

(7) $$\left[ {\matrix{ {{{\rm \tau} _1}} \cr {{{\rm \tau} _2}} \cr } } \right] = {J^T}\left[ {\matrix{ {{M_x}} \cr {{M_y}} \cr } } \right]$$

Where

(8) $${J^{\rm{T}}} = \left[ {\matrix{
   {{l_{11}}\cos {q_{{\rm{d}}1}} + {l_{22}}\cos \left( {{q_{{\rm{d}}1}} - {q_{{\rm{d}}2}}} \right)} & { - {l_{11}}\sin {q_{{\rm{d}}1}} - {l_{22}}\sin \left( {{q_{{\rm{d}}1}} - {q_{{\rm{d}}2}}} \right)}  \cr 
   { - {l_{22}}\cos \left( {{q_{{\rm{d}}1}} - {q_{{\rm{d}}2}}} \right)} & {\sin \left( {{q_{{\rm{d}}1}} - {q_{{\rm{d}}2}}} \right)}  \cr 

 } } \right]$$

Therefore, the experimentalist’s hip joint torque is,

(9) $$\eqalign{{{\rm \tau} _h} = \left( {{l_{11}}\cos{q_{d1}} + {l_{22}}\cos \left( {{q_{d1}} - {q_{ d2}}} \right)} \right){f_x}\left( {{c_y} - {a_y}} \right) \cr\quad + \left( { - {l_{11}}\sin{q_{d2}} - {l_{22}}\sin \left( {{q_{d1}} - {q_{d2}}} \right)} \right){f_y}\left( {{c_x} - {a_x}} \right)}$$

the experimentalist’s knee joint torque is,

(10) $$\eqalign{{{\rm \tau} _k} = - {l_{22}}\cos \left( {{q_{d1}} - {q_{d2}}} \right){f_x}\left( {{c_y} - {a_y}} \right) + \sin \left( {{q_{d1}} - {q_{d2}}} \right){f_y}\left( {{c_x} - {a_x}} \right)}$$

where, ${l_{11}}$ is the length of experimenter’s thigh and ${l_{11}} = 0.4m$. ${l_{22}}$ is the length of experimenter’s calf and ${l_{22}} = 0.38m$.

### Analysis of human movement mechanism

Human walking movement plays a vital role in daily life. The study of human gait parameters has a significant meaning for understanding human movement laws and limbs during walking coordination (Jackson et al., 2004). During walking, the time interval between two consecutive occurrences of the same action in a repetitive motion event is called the gait cycle. Generally speaking, the lower limbs of human body walking cycle is composed of support phase and swing phase (Barbareschi et al., 2015). As with most research results, human trajectory data demonstrate during a normal walking gait cycle, the support phase accounts for about 60% and the swing phase accounts for about 40%. And the proportion of support phase and swing phase in the gait cycle also changes with time.

Human biomechanics is committed to study bone strength, muscle strength, joint range, and various parts speed of the human body. The purpose of research on human biomechanics is to make human-computer interaction play a better role for patients, and avoid doing useless work. Ensure that people wearing LLRR can receive effective rehabilitation treatment and personal safety, and considering the safely and comfort of patients during rehabilitation training. The controller design must be based on the human biomechanics theories, such as the size of the human body's output, the trajectory of the limbs, the smoothness of the movement, and the movement direction of human body parts. In walking process, the clockwise moment required by human joints are found though analysis inverse dynamic. In collected dynamics data, the ground reaction force is the main force that acts on the body during experimenter movement. According to the research needs of this paper, the vertical ground reaction force and the shear force in one direction are studied.

The typical vertical ground reaction force profile of the collected single human walking steps, as shown in Fig. 5. When the heel is in contact with the ground, the vertical ground reaction force is zero and rises sharply within a few tenths of a second. At the moment experimenter’s foot is flat, her weight will dropp and move onto legs. In the middle of standing, the experimenter’s movement center of mass is actually upward. Finally, the experimenter’s feet lost contact with the ground and the force returns to zero when the toes left the ground. It can be seen than the experimenter’s foot force change is a typical M-shaped diagram. Numerous studies have shown that the M-shaped diagram or bimodal diagram are a typical display method of normal gait, which is used to display force fluctuations. The above analysis of the collected experimenter movement data is effective and feasible in this paper.

## Methods

The data collection associated procedures are approved by Zhongyuan University of Technology Committee. The written informed consent was obtained from the participant. And the participant give permission for members of the research team to have access to her human motion data to take part in the research project. In this paper, multiple sets of movement data collected come from a healthy female with a height of 165 cm and a weight of 56 kg. A total of 15 Marker points were attached to both sides of the lower limbs of the experimenter. During the experiment, in order to reduce the influence of external factors on the experiment, experimenter wore black leggings and black sports shoes, and put her hands on the chest and walked naturally parallel.

### Dynamic model of LLRR

**Figure 7:**
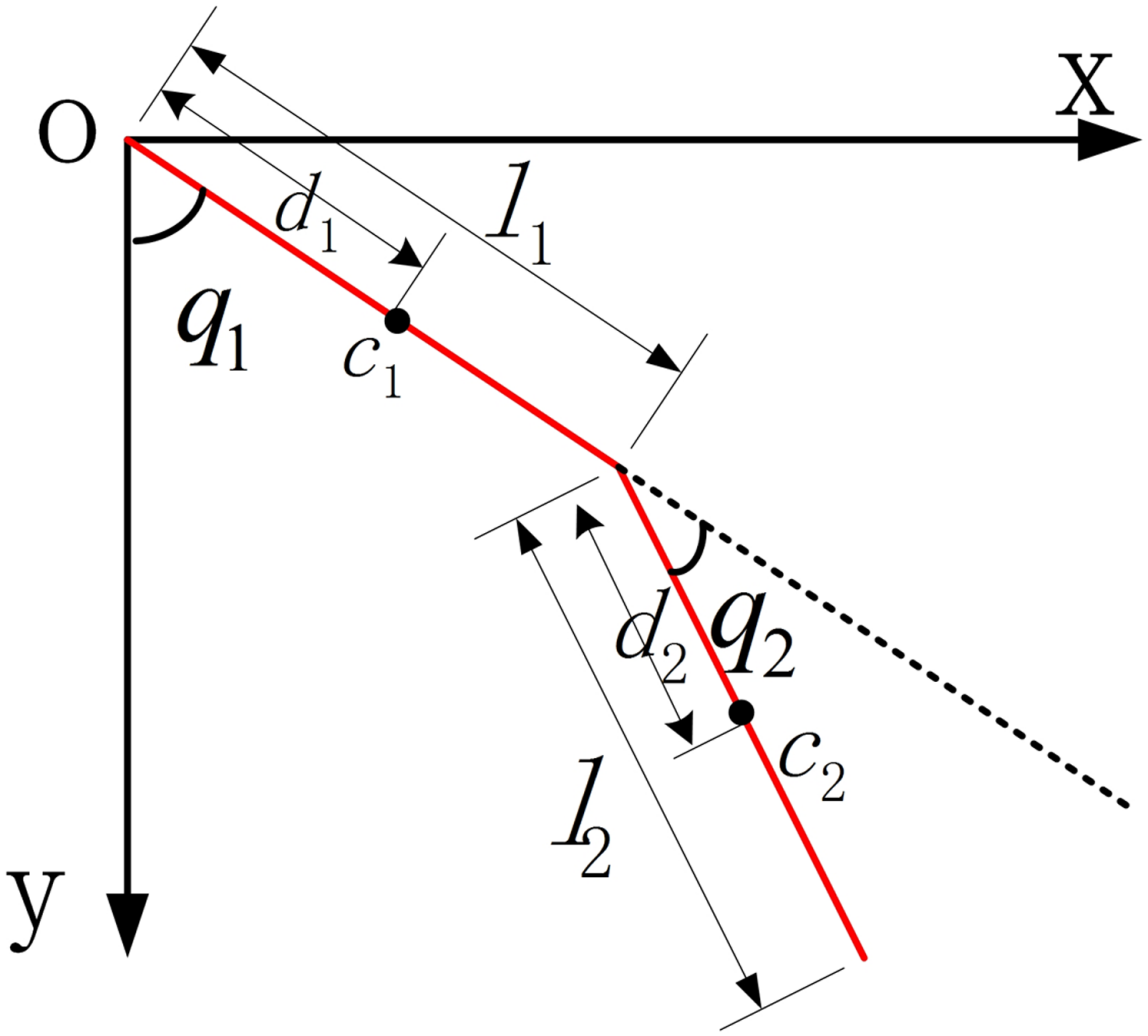
The dynamic model of LLRR.

In this paper, the unilateral leg of LLRR is simplified as a two-bar rigid structure model, as shown in Fig. 7. The Lagrange equation of the LLRR system can be defined as the following form,

(11) $${\tau _1} = \displaystyle{d \over {dt}}\displaystyle{{\partial L} \over {\partial {{\dot q}_1}}} - \displaystyle{{\partial L} \over {\partial {q_1}}} , \quad i = 1,2$$

where, $L$ is the Lagrange function of the LLRR system in the generalized coordinate system, which expressed by the difference between the total kinetic energy and the total potential energy of the LLRR system. ${q_i}$ represent the joint angle, ${\dot q_i}$ represent the joint angle velocity, ${\tau _i}\;$represent the joint torque.

Suppose the thigh length of the LLRR is ${l_1}$, its mass is ${m_1}$, the calf length is ${l_2}$, its mass is ${m_2}$, the center of mass of the thigh rod is ${c_1}$, the center of mass of the calf rod is ${c_2}$, the distance from the hip joint to its center of mass is ${d_1}$, and the distance from the knee joint to its center of mass is ${d_2}$. The angle of the hip joint and the angle of the knee joint of the LLRR are ${q_1}$, ${q_2}$, the joints angular velocity of the LLRR are$\; {\dot q_1}$, ${\dot q_2}$, and the joint angular acceleration are ${\ddot q_1}$, ${\ddot q_2}$, respectively.

The joint torque of the LLRR system can be expressed as,

$${\tau _1} = \displaystyle{d \over {dt}}\displaystyle{{\partial L} \over {\partial {{\dot q}_1}}} - \displaystyle{{\partial L} \over {\partial {q_1}}}$$

$$= \left( {{m_1}d_1^2 + {m_2}l_1^2 + {m_2}d_2^2 + 2{m_2}{l_1}{d_2}\cos{q_2}} \right){\ddot q_1} - \left( {{m_2}{l_1}{d_2}\cos{q_2} + {m_2}d_2^2} \right){\ddot q _2}$$

$$- 2{m_2}{l_1}{d_2}\sin{\dot q_2}{q_2}{\dot q_1} + {m_2}{l_1}{d_2}{\dot q_2}\sin{\dot q_2}{q_2} - {m_2}g{d_2}\sin\left( {{q_1} - {q_2}} \right)$$

(12) $$- {m_1}g{d_1}\sin{q_1} - {m_2}g{l_1}\sin{q_1}$$

$${\tau _2} = \displaystyle{d \over {dt}}\displaystyle{{\partial L} \over {\partial {{\dot q}_2}}} - \displaystyle{{\partial L} \over {\partial {q_2}}}$$

(13) $$= - {m_2}{d_2}\left( {{l_1}\cos{q_2} + {d_2}} \right){\ddot q_1} + {m_2}{l_1}{d_2}\sin{\dot q_2}{q_1} + {m_2}d_2^2{\ddot q_2} + {m_2}g{d_2}\sin\left( {{q_1} - {q_2}} \right)$$

In order to simplify the expression of its equation, make

(14) $$D\left( q \right) = \left[ {\matrix{ {{D_{11}}}  {{D_{12}}} \cr {{D_{21}}}  {{D_{22}}} \cr } } \right] = \left[ {\matrix{ {{m_1}d_1^2 + {m_2}l_1^2 + {m_2}d_2^2 + 2{m_2}{l_1}{d_2}\cos{q_2}} & { - \left( {{m_2}{l_1}{d_2}\cos{q_2} + {m_2}d_2^2} \right)} \cr { - {m_2}{d_2}\left( {{l_1}\cos{q_2} + {d_2}} \right)} & {{m_2}d_2^2} \cr } } \right]$$

(15) $$C\left( q \right) = \left[ {\matrix{ {{C_{11}}} & {{C_{12}}} \cr {{C_{21}}} & {{C_{22}}} \cr } } \right] = \left[ {\matrix{ { - 2{m_2}{l_1}{d_2}{{\dot q}_2}\sin{q_2}} & {{m_2}{l_1}{d_2}{{\dot q}_2}\sin{q_2}} \cr {{m_2}{l_1}{d_2}{{\dot q}_1}\sin{q_2}} & 0 \cr } } \right]$$

(16) $$G\left( q \right) = \left[ {\matrix{ {{G_1}} \cr {{G_2}} \cr } } \right] = \left[ {\matrix{ { - {m_1}g{d_1}\sin{q_1} - {m_2}g{l_1}\sin{q_1} - {m_2}g{d_2}\sin ({q_1} - {q_2})} \cr {{m_2}g{d_2}\sin ({q_1} - {q_2})} \cr } } \right]$$

Equations (12) and (13) can be changed to the following equation,

(17) $$D\left( q \right)\ddot q + C\left( {q,\dot q} \right)\dot q + G\left( q \right) = \tau$$

where, $\tau = {\left[ {\matrix{ {{\tau _1}} & {{\tau _2}} \cr } } \right]^T}$, ${\tau _1}\left( t \right)$ and ${\tau _2}\left( t \right)$ represent the hip joint torque and the knee joint torque of the controller. $D\left( q \right) \in {R^{2 \times 2}}$ represents the inertia matrix, $C\left( {q,\dot q} \right) \in {R^{2 \times 2}}$ represents the Coriolis force and the centripetal force matrix, $G\left( q \right) \in {R^{2 \times 1}}$ represents gravity matrix.

The dynamic characteristics of the LLRR shown in formula Eq. (17) are,

1. $\dot D\left( {\rm\theta} \right) - 2C\left( {{\rm \theta} ,\dot {\rm\theta} } \right)$ is an oblique symmetric matrix.

2. The inertial matrix $D\left( q \right)$ is a $2 \times 2$ symmetric positive definite matrix of. For any $2 \times 1$ vector $\zeta$, there are positive numbers ${m_1}$ and ${m_2}$ satisfying the following inequalities,

$${{m_1}\Vert{\zeta}\Vert^2 \le {\zeta} ^TD\left( q \right)\zeta \le {m_2}\Vert{\zeta}\Vert^2}$$

3. There is a parameter vector $P$ related to the lower limb exoskeleton robot, so that the inertia matrix $D\left( q \right)$, Coriolis force matrix $C\left( q \right)$, and gravity terms $G\left( q \right)$ satisfy the following linear relationships

(19) $$D\left( q \right)\vartheta + C\left( {q,\dot q} \right)\rho + G\left( q \right) = \Phi \left( {q,\dot q,\rho ,\vartheta } \right)P$$

In the formula, $\Phi \left( {q,\dot q,\rho ,\vartheta } \right) \in {R^{2 \times 5}}$ is the regression matrix of the joint variables of the known LLRR. $P \in {R^{5 \times 1}}$ is an unknown fixed-length parameter vector that expresses the characteristics of the robot's mass and inertia.

The RAPLC research on of the LLRR system in this paper, since its controller is designed based on an unknown model, it is necessary to perform online identification of the unknown model. The dynamic characteristics of the robot system are used to separate the known and unknown parameters of the robot model in the identification process. Make

(20) $${p_1} = {m_1}{d_1^2} + {m_2}{l_1^2} + {m_1}{d_2^2}$$

(21) $${p_2} = {m_1}{l_1}{d_2}$$

(22) $${p_3} = {m_2}{d_2^2}$$

(23) $${p_4} = - \left( {{m_1}{d_1} + {m_2}{l_1}} \right)g$$

(24) $${p_5} = {m_2}{d_2}g$$

The Eqs. (14)–(16) can be expressed as,

(25) $$D\left( q \right) = \left[ {\matrix{ {{D_{11}}}  {{D_{12}}} \cr {{D_{21}}}  {{D_{22}}} \cr } } \right] = \left[ {\matrix{ {{p_1} + 2{p_2}\cos{q_2}}  & { - \left( {{p_3} + {p_2}\cos{q_2}} \right)} \cr { - ({p_3} + {p_2}\cos{q_2})}  & {{p_3}} \cr } } \right]$$

(26) $$C\left( {q,\dot q} \right) = \left[ {\matrix{ {{C_{11}}} & {{C_{12}}} \cr {{C_{21}}} & {{C_{22}}} \cr } } \right] = \left[ {\matrix{ { - 2{p_2}{{\dot q}_2}\sin{q_2}} & {{p_2}{{\dot q}_2}\sin{q_2}} \cr {{p_2}{{\dot q}_1}\sin{q_2}} & 0 \cr } } \right]$$

(27) $$G\left( q \right) = \left[ {\matrix{ {{G_1}} \cr {{G_2}} \cr } } \right] = \left[ {\matrix{ {{p_4}\sin{q_1} - {p_5}\sin \left( {{q_1} - {q_2}} \right)} \cr {{p_5}\sin \left( {{q_1} - {q_2}} \right)} \cr } } \right]$$

Substituting Eqs. (25)–(27) into Eq. (19) can be obtained,

(28) $$\left[ {\matrix{ {{D_{11}}} & {{D_{12}}} \cr {{D_{21}}} & {{D_{22}}} \cr } } \right]\left[ {\matrix{ {{\vartheta _1}} \cr {{\vartheta _2}} \cr } } \right] + \left[ {\matrix{ {{C_{11}}} & {{C_{12}}} \cr {{C_{21}}} & {{C_{22}}} \cr } } \right]\left[ {\matrix{ {{\rho _1}} \cr {{\rho _2}} \cr } } \right] + \left[ {\matrix{ {{G_1}} \cr {{G_2}} \cr } } \right] = \Phi \left( {q,\dot q,\rho ,\vartheta } \right)P$$

Then the dynamic equation of the LLRR thigh shaft can be expressed as,

$$\left[ {\matrix{ {{D_{11}}} & {{D_{12}}} \cr } } \right]\left[ {\matrix{ {{\vartheta _1}} \cr {{\vartheta _2}} \cr } } \right] + \left[ {\matrix{ {{C_{11}}} & {{C_{12}}} \cr } } \right]\left[ {\matrix{ {{\rho _1}} \cr {{\rho _2}} \cr } } \right] + {G_1}$$

$$= \left( {{p_1} + 2{p_2}\cos{q_2}} \right){\vartheta _1} - \left( {{p_3} + {p_2}\cos{q_2}} \right){\vartheta _2} - 2{p_2}{\dot q_2}\sin{q_2}{\rho _1} + {p_2}{\dot q_2}\sin{q_2}{\rho _2}$$

$$+ {p_4}\sin{q_1} - {p_5}{\sin}({q_1} - {q_2})$$

$$= {\vartheta _1}{p_1} + \left( {2{\vartheta _1}\cos{q_2} - {\vartheta _2}\cos{q_2}{\vartheta _2}\cos{q_2} - 2{{\dot q}_2}\sin{q_2}{\rho _1} + {{\dot q}_2}\sin{q_2}{\rho _2}} \right){p_2} - {\vartheta _2}{p_3}$$

$$+ \sin{q_1}{p_4} - \sin \left( {{q_1} - {q_2}} \right){p_5}$$

(29) $$= \left[ {\matrix{ {{\Phi _{11}}} & {{\Phi _{12}}} & {{\Phi _{13}}} & {{\Phi _{14}}} & {{\Phi _{15}}} \cr } } \right]\left[ {\matrix{ {{p_1}} \cr {{p_2}} \cr {{p_3}} \cr {{p_4}} \cr {{p_5}} \cr } } \right]$$

where,

$$\; {\Phi _{11}} = {\vartheta _1}$$

$$\; {\Phi _{12}} = 2{\vartheta _1}\cos{q_2} - {\vartheta _2}\cos{q_2}{\vartheta _2}\cos{q_2} - 2{\dot q_2}\sin{q_2}{\rho _1} + {\dot q_2}\sin{q_2}{\rho _2}$$

(30) $$\; {\Phi _{13}} = - {\vartheta _2}$$

$$\; {\Phi _{14}} = \sin{q_1}$$

$$\; {\Phi _{15}} = - \sin \left( {{q_1} - {q_2}} \right)$$

Similarly, the dynamic equation of the LLRR calf rod is,

$$\left[ {\matrix{ {{D_{21}}} & {{D_{22}}} \cr } } \right]\left[ {\matrix{ {{\vartheta _1}} \cr {{\vartheta _2}} \cr } } \right] + \left[ {\matrix{ {{C_{21}}} & {{C_{22}}} \cr } } \right]\left[ {\matrix{ {{{\rm\rho} _1}} \cr {{{\rm\rho} _2}} \cr } } \right] + {G_2}$$

$$= - \left( {{p_3} + {p_2}\cos{q_2}){\vartheta _1} + {p_3}{\vartheta _2} + {p_2}{{\dot q}_1}\sin{q_2}{{\rm\rho} _1} + {p_5}\sin ({q_1} - {q_2}} \right)$$

$$= \left( {{{\dot q}_1}\sin{q_2}{{\rm\rho} _1} - {\vartheta _1}\cos{q_2}){p_2} + \left( {{\vartheta _2} - {\vartheta _1}} \right){p_3} + \sin ({q_1} - {q_2}} \right){p_5}$$

(31) $$= \left[ {\matrix{ {{\Phi _{21}}} & {{\Phi _{22}}} & {{\Phi _{23}}} & {{\Phi _{24}}} & {{\Phi _{25}}} \cr } } \right]\left[ {\matrix{ {{p_1}} \cr {{p_2}} \cr {{p_3}} \cr {{p_4}} \cr {{p_5}} \cr } } \right]$$

where,

$${\Phi _{21}} = 0$$

$${\Phi _{22}} = {\dot q_1}\sin{q_2}{{\rm\rho} _1} - {\vartheta _1}\cos{q_2}$$

(32) $${\Phi _{23}} = {\vartheta _2} - {\vartheta _1}$$

$${\Phi _{24}} = 0$$

$${\Phi _{25}} = \sin ({q_1} - {q_2})$$

Derived from the above formula, the parameters in $\Phi \left( {q,\dot q,{\rm\rho} ,\vartheta } \right)$ are only related to the joint angle and angular acceleration of the LLRR. These data are all known, that is, $\Phi \left( {q,\dot q,{\rm\rho} ,\vartheta } \right)$ is the known parameter of the LLRR system model. $\vartheta$ represents the equation related to acceleration of the end position of the LLRR system. The parameters in $P$ are related to the joint quality, joint length, center of gravity and other data of the LLRR system. However, these parameters in the robot system are often inaccurate or completely unknown, especially the LLRR system. The joint quality is constantly changing real-time when the patient wear it for rehabilitation, so the parameters in $P$ are the unknown parameters.

Assumption 1: ${q_d} \in {R^n}$ is the desired position of joint, ${q_d}$ has first derivative and second derivative.

Assumption 2: The norms of error and disturbance satisfy

$$\Vert\omega\Vert \le {z_1} + {z_2}\Vert{e}\Vert + {z_3}\Vert \dot {e}\Vert$$

where, ${z_1}$,${z_2}$,${z_3}$ are normal numbers, respectively. $e = q - {q_d}$ and $\dot e = \dot q - {\dot q_d}$ are tracking error and tracking error derivative, respectively.

### Controller design

In the rehabilitation process, the comfort of human body wearing LLRR plays a significant role, so this paper proposes a comfort function,

(33) $${\tau _h} = m\dot e + ne = m\left( {{{\dot q}_d} - \dot q} \right) + n\left( {{q_d} - q} \right)$$

where, $m$ and $n$ are positive number. $q$ and $\dot q$ are the angular displacement and angular velocity of the controller, respectively. ${q_d}$ and ${\dot q_{d\; }}$are the angular displacement and angular velocity of the experimenter, respectively.

The unilateral leg dynamics model of the two-link LLRR can be described by Eq. (17), namely,

(34) $$D\left( q \right)\ddot q + C\left( {q,\dot q} \right)\dot q + G\left( q \right) = \tau$$

Introduce variables $y$ and ${q_r}$, and make,

(35) $$y = \dot e + {\rm{\gamma}} e$$

(36) $${q_r} = {\dot q_d} - {{\rm \gamma}} e$$

In the formula ${\rm{\gamma}} > 0$, and

(37) $$y = \dot q - {\dot q_r}$$

In formula Eq. (19) make ${\vartheta} = {\ddot{q} _r}$, ${\rm\rho} = {\dot q_r}$,

(38) $$D\left( q \right){\ddot q _r} + C\left( {q,\dot q} \right){\dot q_r} + G\left( q \right) = \Phi \left( {q,\dot q,{\rm\rho} ,\vartheta } \right)P$$

where,

(39) $${\ddot q _r} = \ddot q - \dot y = \left[ {\matrix{ {{{\ddot q }_1} - \dot y} \cr {{{\ddot q }_2} - \dot y} \cr } } \right] = \left[ {\matrix{ {{{\ddot q }_r}\left( 1 \right)} \cr {{{\ddot q }_r}\left( 2 \right)} \cr } } \right]$$

(40) $${\dot q_r} = q - y = \left[ {\matrix{ {{{\dot q}_1} - y} \cr {{{\dot q}_2} - y} \cr } } \right] = \left[ {\matrix{ {{{\dot q}_r}\left( 1 \right)} \cr {{{\dot q}_r}\left( 2 \right)} \cr } } \right]$$

Substitute formula Eq. (35) into formula Eq. (38),

(41) $$D\left( q \right)\left( {\ddot q - \dot y} \right) + C\left( {q,\dot q} \right)\left( {\dot q - y} \right) + G\left( q \right) = \Phi \left( {q,\dot q,{\rm\rho} ,\vartheta } \right)P$$

Combining formula Eq. (41) and formula Eq. (17) can be obtained,

(42) $$D\left( q \right)\dot y + C\left( {q,\dot q} \right)y = \tau - \Phi \left( {q,\dot q,\rho ,\vartheta } \right)P - \rm\omega$$

For a complex LLRR system, due to the influence of external factors, some errors and deviations will inevitably occur. In order to ensure the LLRR system stability when the upper bound of the disturbance signal is known, the following RAPLC is designed,

(43) $$\tau = \Phi \left( {q,\dot q,{{\dot q}_r},{{\ddot q }_r}} \right)\hat P - {S_p}{K_p}e - {S_v}{K_v}\dot e + u$$

(44) $$D\left( q \right)\dot y + C\left( {q,\dot q} \right)y = \tau - \Phi \left( {q,\dot q,\rho ,\vartheta } \right)P - \rm\omega$$

In the formula Eq. (43),

$${K_p} = {K_{p1}} + {K_{p2}}{B_p}\left( e \right),{K_v} = {K_{v1}} + {K_{v2}}{B_v}\left( {\dot e} \right)\; \; \; \; \; \; \;$$

$${K_{\rm p1}} = {\rm diag}\left( {{k_{\rm p11}},{k_{\rm p12}}} \right),{K_{\rm p2}} = {\rm diag}\left( {{k_{\rm p21}},{k_{\rm p22}}} \right)\; \; \;$$

$${K_{\rm v1}} = {\rm diag}\left( {{k_{\rm v11}},{k_{\rm v12}}} \right),{K_{\rm v2}} = {\rm diag}\left( {{k_{\rm v21}},{k_{\rm v22}}} \right)$$

$${B_p}\left( e \right) = {\rm diag}\left( {\displaystyle{1 \over {{{\rm{\alpha}} _1} + \left| {{e_1}} \right|}},\displaystyle{1 \over {{{\rm{\alpha}} _2} + \left| {{e_2}} \right|}}} \right),{B_v}\left( e \right) = {\rm diag}\left( {\displaystyle{1 \over {{{\rm{\beta}} _1} + \left| {{{\dot e}_1}} \right|}},\displaystyle{1 \over {{{\rm{\beta}} _2} + \left| {{{\dot e}_2}} \right|}}} \right)$$

where, ${{\rm{\gamma}} _1},{{\rm{\gamma}} _2}$ are arbitrarily normal, ${k_{\rm p1i}},{k_{\rm p2i}},{k_{\rm v1i}},{k_{\rm v2i}},{{\rm{\alpha}} _i },{{\rm{\beta}} _i }$ are positive numbers in $i \in \left[ {1,2} \right]$, and satisfy ${k_{\rm p1i}} - \textstyle{{{k_{\rm v2i}}} \over {2{\rm \beta _i }}} > 0$, ${k_{\rm v1i}} - \textstyle{{{k_{\rm p2i}}} \over {2{\rm \alpha _i }}} > 0$. ${S_p}$ and ${S_v}$ are S-shaped function matrix, and,

${S_p} = \left[ {\matrix{ {\displaystyle{{{c_1}} \over {1 + {e\rm ^{ - {a_1}t}}}}}  0 \cr 0  {\displaystyle{{{c_2}} \over {1 + {e\rm ^{ - {a_2}t}}}}} \cr } } \right]$, ${S_v} = \left[ {\matrix{ {\displaystyle{1 \over {1 + {e\rm ^{ - {b_1}t}}}}} & 0 \cr 0 & {\displaystyle{1 \over {1 + {e\rm ^{ - {b_2}t}}}}} \cr } } \right]$

where, ${a_1},{a_2},{b_1},{b_2}$ are the decay factor of the exponential function, ${c_1}$and ${c_2}$ are the coefficients of the exponential function.

The parameter estimation law of $\hat P$,

(45) $$\overset{.}{\mathop{{\overset{\lower0.5em\hbox{$\smash{\scriptscriptstyle\frown}$}}{P}}}}\,=-\text{ }\!\!\Gamma\!\!\text{ }{{\text{ }\!\!\Phi\!\!\text{ }}^{T}}\left( q,\dot{q},{{{\dot{q}}}_{r}},{{\overset{\ddot{\ }}{\mathop{q}}\,}_{r}} \right)y$$

where, $\Gamma$ is a symmetric positive definite matrix. For the LLRR system shown in Eq. (17), and when the disturbance error signal is bounded, the designed control method can ensure the global gradual stability of the system.

### Stability analysis

Make $\tilde P = \dot P - P$, and take the Lyapunov function of LLRR as,

(46) $$V = \displaystyle{1 \over 2}\left( {{y^T}D\left( q \right)y + {e^T}\left( {{S_p}{K_{\rm p1}} + {\rm{\gamma}} {S_v}{K_{\rm v1}}} \right)e + {{\tilde P}^T}{\Gamma ^{ - 1}}\tilde P} \right)$$

According to the dynamic characteristics of the LLRR, $D\left( q \right)$ is a symmetric positive definite matrix, ${S_p}$ and ${S_v}$ are also symmetric positive definite matrix. So,

(47) $${\left( {{y^T}D\left( q \right)y} \right)^{\rm '}} = 2{y^T}D\left( q \right)\dot y + {y^T}\dot D\left( q \right)y$$

(48) $${\left( {{e^T}\left( {{S_p}{K_{\rm p1}} + {\rm{\gamma}} {S_v}{K_{\rm v1}}} \right)e} \right)^{'}} = 2{e^T}\left( {{S_p}{K_{\rm p1}} + {\rm{\gamma}} {S_v}{K_{\rm v1}}} \right)\dot e$$

(49) $${\left( {{{\tilde P}^T}{\Gamma ^{ - 1}}\tilde P} \right)^{'}} = 2{\tilde P^T}{\Gamma ^{ - 1}}{\dot{\tilde{P}}}$$

Take the derivative of Eq. (46) to get,

$$\dot V = {y^T}D\left( q \right)\dot y + \displaystyle{1 \over 2}{y^T}\mathop {D\left( q \right)}\limits^\dot y{e^T} + {e^T}\left( {{S_p}{K_{\rm p1}} + {\rm{\gamma}} {S_v}{K_{\rm v1}}} \right)\dot e + {\tilde P^T}{\Gamma ^{ - 1}}{\dot{\tilde{P}}}$$

$$= - {\dot e^T}{S_v}{K_v}\dot e - {\rm{\gamma}} {e^T}{S_p}{K_p}e - {e^T}\left( {{S_p}{K_{p2}}{B_p}\left( e \right) + {\rm{\gamma}} {S_v}{K_{v2}}{B_p}\left( {\dot e} \right)} \right)\dot e$$

(50) $$+ {\tilde P^T}{\Gamma ^{ - 1}}\mathop {\tilde P}\limits^\dot + {y^T}\Phi \left( {q,\dot q,{{\dot q}_r},{{\ddot q }_r}} \right)\tilde P + {y^T}\left( {u - \omega } \right)$$

Combine ${y^T}\Phi \left( {q,\dot q,{{\dot q}_r},{{\ddot q }_r}} \right)\tilde P = {\tilde P^T}{\Phi ^T}\left( {q,\dot q,{{\dot q}_r},{{\ddot q }_r}} \right)y$, ${\dot{\tilde{P}}} = {\dot{\hat{P}}}$ and Eq. (45),

(51) $${y^T}\Phi \left( {q,\dot q,{{\dot q}_r},{{\ddot q }_r}} \right)\tilde P + {\tilde P^T}{\Gamma ^{ - 1}}{\dot{\tilde P}} = 0$$

Then,

$$\dot V = - {\dot e^T}{S_v}{K_v}\dot e - {\rm{\gamma}} {e^T}{S_p}{K_p}e - {e^T}\left( {{S_p}{K_{\rm p2}}{B_p}\left( e \right) + {\rm{\gamma}} {S_v}{K_{\rm v2}}{B_p}\left( {\dot e} \right)} \right)\dot e + {y^T}\left( {u - \omega } \right)$$

$$= - \mathop \sum \limits_{i = 1}^2 \left( {{\rm{\gamma}} \left( {{S_p}{k_{p1i}} + \displaystyle{{{S_p}{k_{p2i}}} \over {{{\rm{\alpha}} _i} + |{e_i}|}}} \right){e_i}^2} \right) - \mathop \sum \limits_{i = 1}^2 \left( {\left( {{S_v}{k_{v1i}} + \displaystyle{{{S_v}{k_{v2i}}} \over {{{\rm{\beta}} _i} + \left| {\dot{{e_i}}} \right|}}} \right){{\dot{{e_i}}}^2}} \right) $$

(52) $$- \mathop \sum \limits_{i = 1}^2 \left( {\left( {\displaystyle{{{S_p}{k_{p2i}}} \over {{{\rm{\alpha}} _i} + |{e_i}|}} + \displaystyle{{{\rm{\gamma}} {S_v}{k_{v2i}}} \over {{{\rm{\beta}} _i} + \left| {\dot{{e_i}}} \right|}}} \right){e_i}\dot{{e_i}}} \right) + {y^T}\left( {u - \omega } \right)$$

The following formula can be obtained due to ${e_i}{\dot e_i} \le \displaystyle{1 \over 2}\left( {{e_i}^2 + {{\dot e}_i}^2} \right)$,

$$\dot V \le - \mathop \sum \limits_{i = 1}^2 \left( {{\rm{\gamma}} \left( {{S_p}{k_{p1i}} + \displaystyle{{{S_p}{k_{p2i}}} \over {{{\rm{\alpha}} _i} + |{e_i}|}}} \right) - \displaystyle{1 \over 2}\left( {\displaystyle{{{S_p}{k_{p2i}}} \over {{{\rm{\alpha}} _i} + |{e_i}|}} + \displaystyle{{{\rm{\gamma}} {S_v}{k_{v2i}}} \over {{{\rm{\beta}} _i} + \left| {\dot{e}_i} \right|}}} \right)} \right){e_i}^2$$

(53) $$- \mathop \sum \limits_{i = 1}^2 \left( {\left( {{S_v}{k_{v1i}} + \displaystyle{{{S_v}{k_{v2i}}} \over {{{\rm{\beta}} _i} + \left| {\dot{{e_i}}} \right|}}} \right) - \displaystyle{1 \over 2}\left( {\displaystyle{{{S_p}{k_{p2i}}} \over {{{\rm{\alpha}} _i} + |{e_i}|}} + \displaystyle{{{\rm{\gamma}} {S_v}{k_{v2i}}} \over {{{\rm{\beta}} _i} + \left| {\dot{{e_i}}} \right|}}} \right){{\dot{{e_i}}}^2}} \right) + {y^T}\left( {u - \omega } \right)$$

When $\displaystyle{1 \over 2} \le {\rm{\gamma}} \le 2$,

$${\rm{\gamma}} \displaystyle{{{S_p}{k_{\rm p2i}}} \over {{{\rm{\alpha}} _i } + \left| {{e_i }} \right|}} - \displaystyle{1 \over 2}\displaystyle{{{S_p}{k_{\rm p2i}}} \over {{{\rm{\alpha}} _i } + \left| {{e_i }} \right|}} \ge 0$$

(54) $$\displaystyle{{{S_v}{k_{\rm v2i}}} \over {{{\rm{\beta}} _i } + \left| {{{\dot e}_i }} \right|}} - \displaystyle{1 \over 2}\displaystyle{{{\rm{\gamma}} {S_v}{k_{\rm v2i}}} \over {{{\rm{\beta}} _i } + \left| {{{\dot e}_i }} \right|}} \ge 0$$

Formula Eq. (55) can be expressed,

$$\dot{V} \le - \mathop \sum \limits_{i = 1}^2 \left( {{\rm{\gamma}} \left( {{S_p}{k_{p1i}} - \displaystyle{1 \over 2}\displaystyle{{{S_v}{k_{v2i}}} \over {{{\rm{\beta}} _i} + \left| {\dot{e}_i} \right|}}} \right){e_i^2} + \left( {{S_v}{k_{v1i}} - \displaystyle{1 \over 2}\displaystyle{{{S_p}{k_{p2i}}} \over {{{\rm{\alpha}} _i} + |{e_i}|}}} \right)} \right) + {y^T}\left( {u - \rm \omega } \right)$$

$$\le - \mathop \sum \limits_{\rm i = 1}^2 \left( {{\rm{\gamma}} \left( {{S_p}{k_{\rm p1i}} - \displaystyle{1 \over 2}\displaystyle{{{S_v}{k_{\rm v2i}}} \over {{{\rm{\beta}} _i}}}} \right){e_i ^2} + \left( {{S_v}{k_{\rm v1i}} - \displaystyle{1 \over 2}\displaystyle{{{S_p}{k_{\rm p2i}}} \over {{{\rm{\alpha}} _i }}}} \right)} \right) + {y^T}\left( {u - \rm\omega } \right)$$

(55) $$\le {y^T}\left( {u - \rm\omega } \right)$$

Since,

$${y^T}u = \mathop \sum \limits_{\rm i = 1}^2 {y_i }\left[ { - \left( {{z_1} + {z_2}e + {z_3}\dot e} \right){\rm sgn}\left( {{y_i }} \right)} \right]$$

$$\le \mathop \sum \limits_{\rm i = 1}^2 - \left( {||\omega || . ||{y_i }||} \right){\rm sgn}\left( {{y_i }} \right) - {y^T}\rm\omega$$

(56) $$\le ||{y^T}||. ||\rm\omega||$$

Then,

(57) $${y^T}\left( {u - \rm\omega } \right) \le \mathop \sum \limits_{\rm i = 1}^2 \left( { - ||{\rm\omega}|| \cdot ||{y_i }||} \right) + ||{y^T}|| \cdot ||\rm\omega|| = 0$$

Namely,

(58) $$\dot V \le 0$$

It can be seen from the above proof results, $\mathop {lim}\nolimits_{t \to \infty } e = 0$, $\mathop {lim}\nolimits_{t \to \infty } \dot e = 0$, the LLRR control system is globally asymptotically stable. Therefore, the LLRR system can realize the desired trajectory tracking no matter where it starts from the initial position.

### Comfort evaluation function

When the human body wears the LLRR, too much fluctuation of the angle of the system will cause discomfort to the wearer. The comfort of patients is usually known through subjective feelings, but most patients with lower limb movement disorders are not sensitive. Most studies usually evaluate changes in angle, joint torque, changes in patient muscle strength, etc. This paper uses the similarity formula Eq. (59) between the trajectory of the lower limb exoskeleton robot and the trajectory of the human body to objectively evaluate the comfort of the human body wearing LLRR.

(59) $$C = \displaystyle{1 \over {1 + {{\bigg(\displaystyle{{{q_1} - {q_{\rm d1}}} \over {{q_{\rm 1max}} - {q_{\rm d1min}}}}\bigg)}^2} + {{\bigg(\displaystyle{{{q_2} - {q_{\rm d2}}} \over {{q_{\rm 2max}} - {q_{\rm d2min}}}}\bigg)}^2}}}$$

where, ${q_1}\;$and ${q_{\rm d1}}$ are LLRR hip joint and experimenter hip joint. ${q_2}$ and ${q_{\rm d2}}$ are LLRR knee joint and experimenter knee joint. ${q_{\rm 1max}}\;$ and ${q_{\rm 2max}}$ are the maximum values of LLRR hip and knee joints. ${q_{\rm d1min}}$ and ${q_{\rm d2min}}$ are the minimum values of the hip and knee joints of experimenter. When *C* is close to 1, it means that LLRR trajectory is very similar to the trajectory of the human body, which indirectly reflects that the comfort of the human body wearing LLRR will be higher.

## Results

In the second section of this paper, the hip joint and knee joint trajectories are obtained after data collection, processing, and fitting as the reference trajectories of the LLRR. Assume that the initial state of the LLRR is zero. And the acceleration of gravity is 9.8 m/s.

Make ${z_1} = 2$, ${z_2} = 3$, ${z_3} = 6$ and assume that the initial value of the error perturbation is,

$$\omega = 0.05 + 0.05t\sin\left( {2\pi t} \right)\;$$

RAPLC parameters are designed as,

$a = 0.2$, $b = 1$

${\rm {\rm{\alpha}} _i} = 1$, ${\rm \beta _i} = 1\left( {\rm i = 1,2} \right)$, ${\rm{\gamma}} = 5$, $\Gamma = {\rm diag}\left( {5,5,5} \right)$

${K_{\rm p1}} = {\rm diag}\left( {50,50} \right)$,$\; {K_{\rm p2}} = {\rm diag}\left( {130,130} \right)$

${K_{\rm v1}} = {\rm diag}\left( {100,100} \right)$,$\; {K_{\rm v2}} = {\rm diag}\left( {100,100} \right)$

${S_p} = \left[ {\matrix{ {\displaystyle{{80} \over {1 + {e^{\rm - 100t}}}}} & 0 \cr 0 & {\displaystyle{{40} \over {1 + {e^{\rm - 100t}}}}} \cr } } \right]$, ${S_v} = \left[ {\matrix{ {\displaystyle{1 \over {1 + {e^{\rm - 100t}}}}} & 0 \cr 0 & {\displaystyle{1 \over {1 + {e^{\rm - 100t}}}}} \cr } } \right]$

**Figure 8:**
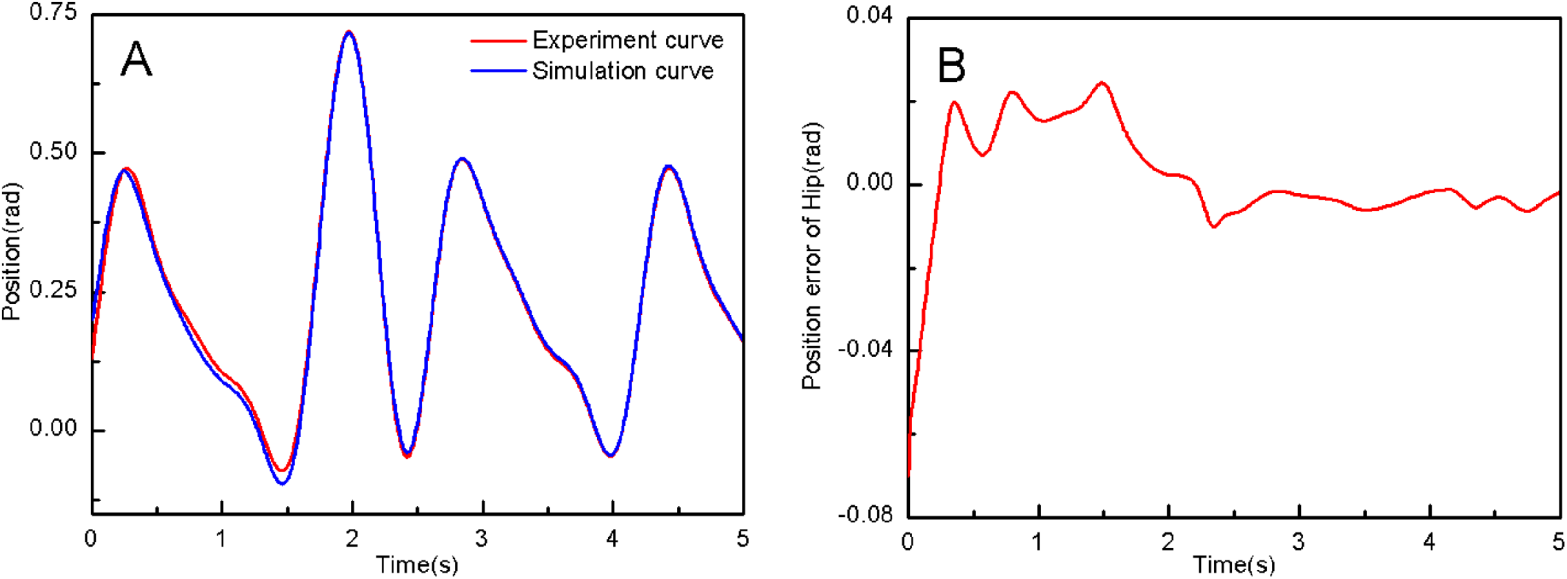
(A) The hip joint position tracking based on RAPC. (B) Position error of hip.

**Figure 9:**
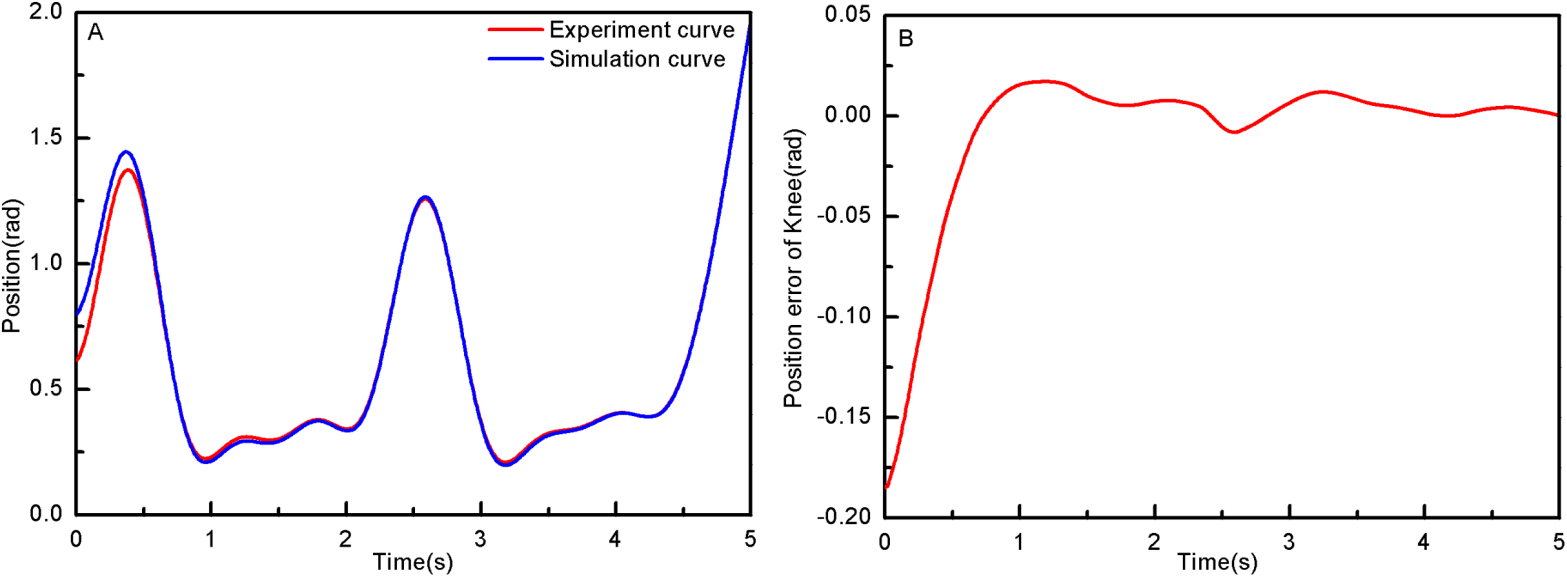
(A) The knee joint position tracking based on RAPC. (B) Position error of knee.

**Figure 10:**
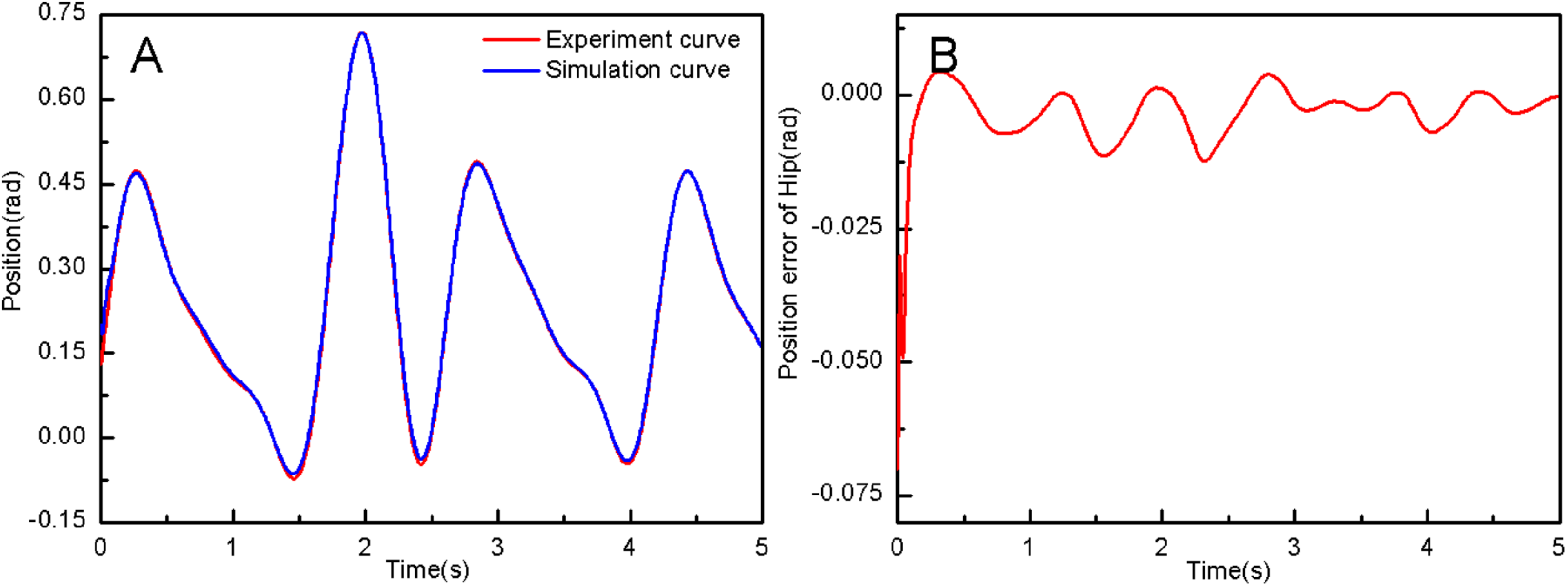
(A) The hip joint position tracking based on RAPDC. (B) Position error of hip.

**Figure 11:**
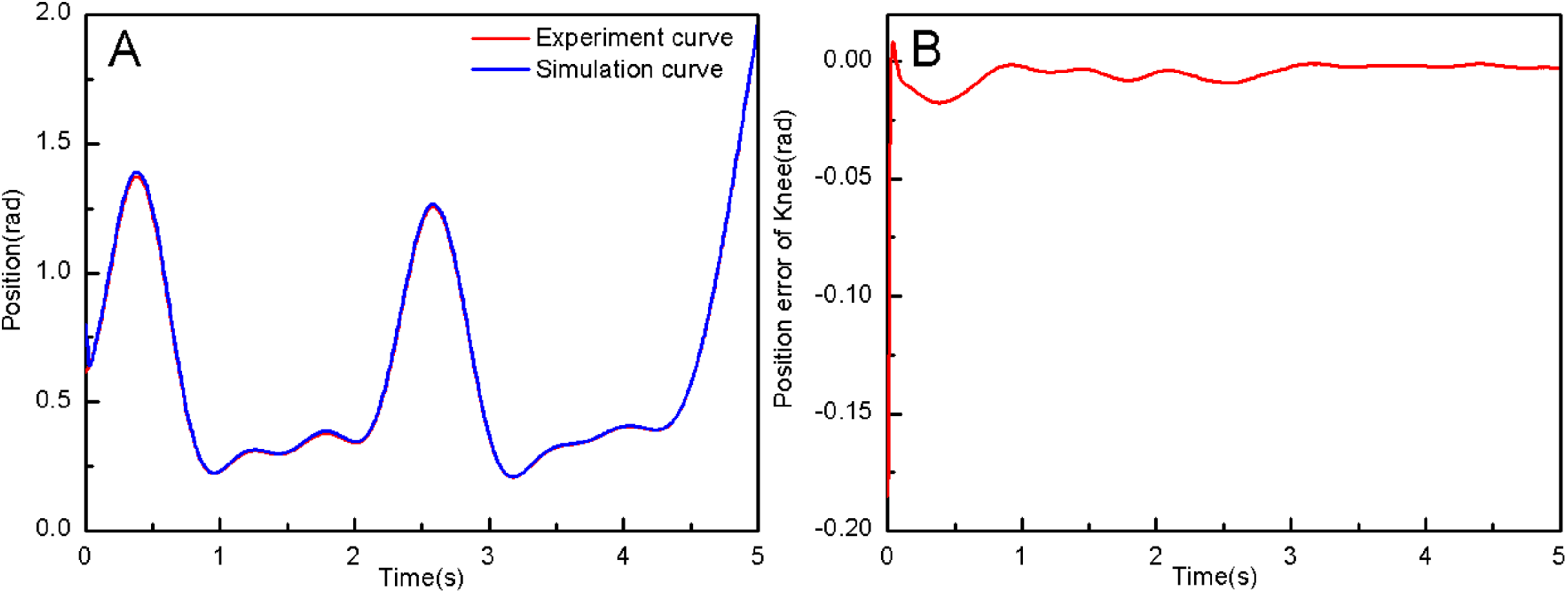
(A) The knee joint position tracking based on RAPDC. (B) Position error of knee.

**Figure 12:**
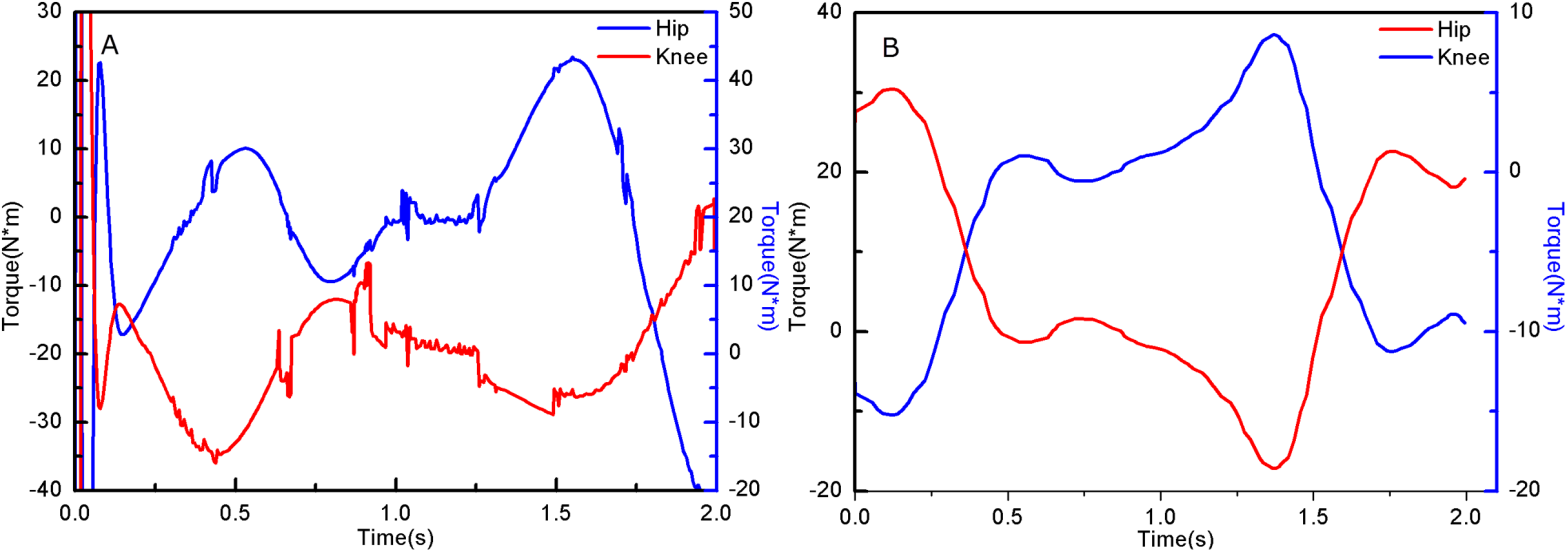
(A) Hip and knee joint torque of simulation data. (B) Hip and knee joint torque of experiment data.

**Figure 13:**
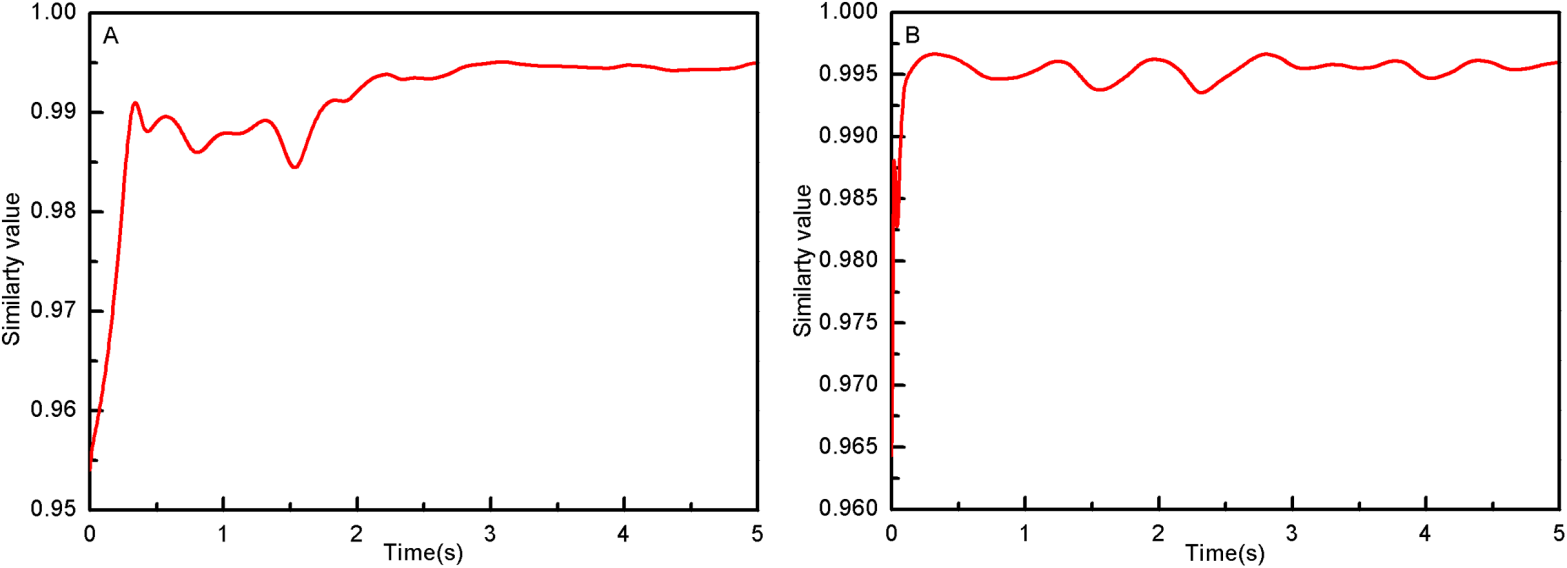
(A) The joint similarity curve of RAPC. (B) The joint similarity curve of RAPDC.

From the simulation diagram of the LLRR control system based on the experimental data, it can be seen that Fig. 8A shows the hip joint trajectory tracking curve of the robust adaptive PD control (RAPC) based on the experimental data. And the hip joint angular displacement starts to converge at 2.6 s and the convergence error is 1% in Fig. 8B. Figure 9A shows the knee joint trajectory tracking curve of RAPC, and knee joint angular displacement starts to converge at 1.132 S with an error of 1% in Fig. 9B. Figure 10A shows the hip joint trajectory tracking curve of the RAPDC based on the experimental data. The hip joint angular displacement can achieve a convergence error of 1% within 0.1655 s in Fig. 10B. Figure 11A shows the hip and knee joint trajectory tracking curves of the RAPDC. Figure 11B can achieve a convergence error of 0.1% within 0.048 s of the knee joint angular displacement. It can be proved that RAPDC has a better performance by comparing the joints trajectory tracking convergence speed of RAPC and RAPDC. The joints angle of the LLRR system can track the gait trajectory of a normal person so quickly with errors and interferences, and the joints position tracking error quickly converges within the ideal range, the S-curve function plays an essential role. Figure 12A shows the hip and knee joints torque curves of the RAPDC based on experiment data, the joint input torque of the robot is relatively small. Compared with the collected real torque of the human body (Fig. 12B), it is found that it has the same movement trend as the real torque curve and the torque range of the experiment data and simulation result is different, Fig. 13A is higher, because LLRR system must provide motivation for Human-robot. And the small fluctuations come from external interference, which proves that the designed controlled has better robust performance. The hip and knee joint torque are in Fig. 13B only from humans. The above results demonstrate the method proposed in the paper is effective and feasible. Figure 13A shows the joint similarity curve based on comfort function of RAPC and the curve can be stabilized near 0.995 within 2.5 s. Figure 13B shows the joint similarity curve based on comfort function of RAPLC. it can reach a higher similarity faster compared with Fig. 13A and the curve can quickly stabilize around 0.995 within 0.048 s and the fluctuation range is within the allowable error. It can be found that compared with RAPC, RAPLC can ensure that patients wear LLRR for a rehabilitation training in an extremely short time, which is very important for patients. The extremely high comfort can objectively indicate that the patient can perform more comfortable rehabilitation training, and the proposed method has a better control effect of the LLRR system in this paper.

## Conclusions

In this article, the research innovations are summarized as follows:

(1) The experimental data collected from healthy humans was used as the reference expected trajectory of the LLRR control system, and this paper analyzes its dynamics and operational analysis based on human movement characteristics.

(2) Considering comfort, tracking convergence speed, and others, a RAPLC was designed based on human movement mechanism. The controller introduced an S-curve function and comfort formula to improves the convergence speed of the joint angular displacement and reduces the joint initial torque. The results show that this method has a faster convergence rate and a lower input torque than RAPC.

(3) Simulation verification of the lower extremity exoskeleton robot control system based on experimental data. The joint torque obtained by simulation is very similar to the collected real human joint torque, which shows that the method designed in this paper is effective and feasible. It is verified by the similarity function that this paper proposed can ensure the patient has a higher level of comfort when wearing LLRR.

## Supplemental Information

10.7717/peerj-cs.394/supp-1 Supplemental Information 1 Raw data.

## Additional Information and Declarations

### Data Availability

The following information was supplied regarding data availability: Raw data, including human movement data, is available as a Supplemental File.

## References

[ref-1] Abd AT, Singh RE, Iqbal K, White G (2020). Muscle synergies are robust across participants in upper limb rotational motion.

[ref-2] Ariizumi R, Takahashi R, Tanaka M, Asai T (2019). Head-trajectory-tracking control of a snake robot and its robustness under actuator failure. IEEE Transactions on Control Systems Technology.

[ref-3] Badesa FJ, Morales R, Garcia-Aracil NM, Sabater JM, Zollo L, Papaleo E, Guglielmelli E (2016). Dynamic adaptive system for robot-assisted motion rehabilitation. IEEE Systems Journal.

[ref-4] Barbareschi G, Richards R, Thornton M, Carlson T, Holloway C (2015). Statically vs dynamically balanced gait: Analysis of a robotic exoskeleton compared with a human.

[ref-5] Becerra FAG, Ortega AB, Beltran CDG, Valdivia CG, Arcega ROD (2018). Design and control of a new parallel robot for the rehabilitation of the hip-knee. IEEE Latin America Transactions.

[ref-6] Boudjedir CE, Boukhetala D, Bouri D (2018). Nonlinear PD control of a parallel delta robot expermentals results.

[ref-7] Cao J, Zhang J, Zhu H (2017). Nonlinear compensation control for high-speed industrial robot.

[ref-8] Celentano L, Basin MV (2020). An approach to design robust tracking controllers for nonlinear uncertain systems. IEEE Transactions on Systems, Man, and Cybernetics: Systems.

[ref-9] Chen Q, Zi B, Sun Z, Li Y, Xu Q (2019). Design and development of a new cable-driven parallel robot for waist rehabilitation. IEEE/ASME Transactions on Mechatronics.

[ref-10] Cheng L, Lin Y, Hou Z-G, Tan M, Huang J, Zhang WJ (2011). Adaptive tracking control of hybrid machines: a closed-chain five-bar mechanism case. IEEE/ASME Transactions on Mechatronics.

[ref-11] Cheng L, Lin Y, Hou Z-G, Tan M, Huang J, Zhang WJ (2012). Integrated design of machine body and control algorithm for improving the robustness of a closed-chain five-bar machine. IEEE/ASME Transactions on Mechatronics.

[ref-12] Dong Y, Ren B (2019). UDE-based variable impedance control of uncertain robot systems. IEEE Transactions on Systems, Man, and Cybernetics: Systems.

[ref-13] Ivanov V, Zhilenkova EA (2019). Software environment for motion capture system based on inertial sensors.

[ref-14] Jackson BD, Wluka AE, Teichtahl AJ, Morris ME, Cicuttini FM (2004). Reviewing knee osteoarthritis—a biomechanical perspective. Journal of Science and Medicine in Sport.

[ref-15] Jiang TT, Qian ZQ, Lin Y, Bi ZM, Liu YF, Zhang WJ (2017). Analysis of virtual environment haptic robotic systems for a rehabilitation of post-stroke patients.

[ref-16] Kim W-S, Cho S, Ku J, Kim Y, Lee K, Hwang H-J, Paik N-J (2020). Clinical application of virtual reality for upper limb motor rehabilitation in stroke: review of technologies and clinical evidence. Journal of Clinical Medicine.

[ref-17] Kim A, Lee YS (2019). Application of sliding rehabilitation machine in patients with severe cognitive dysfunction after stroke. Applied Sciences.

[ref-18] Kong L, He W, Yang C, Li Z, Sun C (2019). Adaptive fuzzy control for coordinated multiple robots with constraint using impedance learning. IEEE Transactions on Cybernetics.

[ref-19] Kong X, Majumdar H, Zang F, Jiang S, Wu Q, Zhang W (2018). A multi-switching mode intelligent hybrid control of electro-hydraulic proportional systems. Proceedings of the Institution of Mechanical Engineers, Part C: Journal of Mechanical Engineering Science.

[ref-20] Kurihara K, Hoshino S, Yamane K, Nakamura Y (2002). Optical motion capture system with pan-tilt camera tracking and real time data processing. IEEE International Conference on Robotics and Automation.

[ref-21] Lee J, Park SH, Chang PH, Suh J, Seo K-H, Jin M (2019). Improved adaptive PID control using time-delay estimation for robot manipulators.

[ref-22] Li Z, Xiao S, Ge SS, Su H (2016). Constrained multilegged robot system modeling and fuzzy control with uncertain kinematics and dynamics incorporating foot force optimization. IEEE Transactions on Systems, Man, and Cybernetics: Systems.

[ref-23] Ling S, Wang H, Liu PX (2020). Adaptive fuzzy tracking control of flexible-joint robots based on command filtering. IEEE Transactions on Industrial Electronics.

[ref-24] Liu D-X, Wu X, Du W, Wang C, Chen C, Xu T (2017). Deep spatial-temporal model for rehabilitation gait: optimal trajectory generation for knee joint of lower-limb exoskeleton. Assembly Automation.

[ref-25] Pfister A, West AM, Bronner S, Noah JA (2014). Comparative abilities of Microsoft Kinect and Vicon 3D motion capture for gait analysis. Journal of Medical Engineering & Technology.

[ref-26] Ren H, Liu D-X, Li N, He Y, Yan Z, Wu X (2018). On-line dynamic gait generation model for wearable robot with user’s motion intention.

[ref-27] Saeed MT, Qin S (2019). Robust control of a mechatronic exoskeleton for motion rehabilitatio.

[ref-28] Wang F, Qian Z, Lin Y, Zhang W (2020). Design and rapid construction of a cost-effective virtual haptic sevice. IEEE/ASME Transactions on Mechatronics.

[ref-29] Xia G, Sun H, Niu X, Zhang G, Feng L (2017). Keyframe extraction for human motion capture data based on joint kernel sparse representation. IEEE Transactions on Industrial Electronics.

[ref-30] Xue T, Wang Z, Zhang T, Zhang M (2019). Adaptive oscillator-based robust control for flexible hip assistive exoskeleton. IEEE Robotics and Automation Letters.

[ref-31] Yang Y, Dong X, Liu X, Huang D (2020). Robust repetitive learning-based trajectory tracking control for a leg exoskeleton driven by hybrid hydraulic system. IEEE Access.

[ref-32] Yang C, Lin Y, Cai M, Qian Z, Kivol J, Zhang W (2017). Cognitive fatigue effect on rehabilitation task performance in a haptic virtual environment system. Journal of Rehabilitation and Assistive Technologies Engineering.

[ref-33] Yin X, Zhang Q, Wang H, Ding Z (2019). RBFNN-based minimum entropy filtering for a class of stochastic nonlinear systems. IEEE Transactions on Automatic Control.

[ref-34] Zeng Y, Dai H, Zheng M, Su S, Wu Z, Xia X, Lin Z, Wu Q (2016). A 3D passive optical localization system based on binocular infrared cameras.

[ref-35] Zhang Y, Cao J (2015). 3D human motion key-frames extraction based on asynchronous learning factor PSO.

[ref-36] Zhang Q-C, Hu L, Gow J (2020). Output feedback stabilization for MIMO semi-linear stochastic systems with transient optimisation. International Journal of Automation and Computing.

[ref-37] Zhang Q, Zhou J, Wang H, Chai T (2016). Output feedback stabilization for a class of multi-variable bilinear stochastic systems with stochastic coupling attenuation. IEEE Transactions on Automatic Control.

[ref-38] Zhou F, Yang S, Fujita H, Chen D, Wen C (2020). Deep learning fault diagnosis method based on global optimization GAN for unbalanced data. Knowledge-Based Systems.

